# Reactive oxygen species‐responsive mitochondria‐targeted liposomal quercetin attenuates retinal ischemia–reperfusion injury via regulating SIRT1/FOXO3A and p38 MAPK signaling pathways

**DOI:** 10.1002/btm2.10460

**Published:** 2022-12-01

**Authors:** Laien Zhao, Longbing Ling, Jing Lu, Feng Jiang, Jianchao Sun, Zhen Zhang, Yanmei Huang, Xiaoqian Liu, Yanjuan Zhu, Xiaoxuan Fu, Shengjun Peng, Wenze Yuan, Ruikang Zhao, Zhuhong Zhang

**Affiliations:** ^1^ School of Pharmacy, Key Laboratory of Molecular Pharmacology and Drug Evaluation (Yantai University), Ministry of Education, Collaborative Innovation Center of Advanced Drug Delivery System and Biotech Drugs in Universities of Shandong Yantai University Yantai People's Republic of China; ^2^ Department of Ophthalmology Tianjin Medical University General Hospital Tianjin People's Republic of China; ^3^ School of Environment and Material Engineering Yantai University Yantai People's Republic of China; ^4^ College of Chemistry and Chemical Engineering Yantai University Yantai People's Republic of China

**Keywords:** FOXO3A, mitochondria targeted, quercetin, retinal ischemia–reperfusion injury, ROS‐responsive liposomes

## Abstract

Retinal ischemia–reperfusion (RIR) injury is involved in the pathogenesis of various vision‐threatening diseases. The overproduction of reactive oxygen species (ROS) is thought to be the main cause of RIR injury. A variety of natural products, including quercetin (Que), exhibit potent antioxidant activity. However, the lack of an efficient delivery system for hydrophobic Que and the presence of various intraocular barriers limit the effective retinal delivery of Que in clinical settings. In this study, we encapsulated Que into ROS‐responsive mitochondria‐targeted liposomes (abbreviated to Que@TPP‐ROS‐Lips) to achieve the sustained delivery of Que to the retina. The intracellular uptake, lysosome escape ability, and mitochondria targeting ability of Que@TPP‐ROS‐Lips were evaluated in R28 retinal cells. Treating R28 cells with Que@TPP‐ROS‐Lips significantly ameliorated the decrease in ATP content, ROS generation, and increase in the release of lactate dehydrogenase in an in vitro oxygen–glucose deprivation (OGD) model of retinal ischemia. In a rat model, the intravitreal injection of Que@TPP‐ROS‐Lips 24 h after inducing retinal ischemia significantly enhanced retinal electrophysiological recovery and reduced neuroinflammation, oxidative stress, and apoptosis. Que@TPP‐ROS‐Lips were taken up by retina for at least 14 days after intravitreal administration. Molecular docking and functional biological experiments revealed that Que targets FOXO3A to inhibit oxidative stress and inflammation. Que@TPP‐ROS‐Lips also partially inhibited the p38 MAPK signaling pathway, which contributes to oxidative stress and inflammation. In conclusion, our new platform for ROS‐responsive and mitochondria‐targeted drug release shows promise for the treatment of RIR injury and promotes the clinical application of hydrophobic natural products.

## 1 INTRODUCTION

Retinal ischemia–reperfusion (RIR) injury is involved in the pathogenesis of various vision‐threatening diseases such as central retinal artery occlusion, ocular ischemic syndrome, diabetic retinopathy (DR), and glaucoma. By 2020, glaucoma, DR, and age‐related macular degeneration (AMD) collectively caused more than 19 million cases of moderate or severe visual impairment in adults aged 50 and older.^1^ Therefore, new preventive and therapeutic strategies for these ocular diseases are urgently needed. However, the eye has multiple biological barriers, including tear film barrier, cornea barrier, conjunctiva and the blood–retinal barrier, and the barriers of posterior segment of the eye consist of the tight junctions present in the ciliary body epithelium, endothelial cells of the iris, retinal pigment epithelium (RPE), and the retina.^2^, ^3^ These physiological barriers pose a challenge in the treatment of posterior segment eye diseases. Retinal disease is difficult to be treated systemic or periocular routes of administration.^4^ Conventional treatments mainly include surgery, laser treatment, eye drops, and intraocular injections (e.g., anti‐VEGF) to inhibit disease progression and bypass the barriers bypass barriers as possible.^5^ However, traditional drugs suffer from poor permeation, ineffective drug distribution, insufficient drug bioavailability, and poor long‐term therapeutic effectiveness, resulting in the need for repeated intravitreal intervention. Therefore, new treatments for retina diseases are needed to improve patient compliance, enhance biocompatibility, and reduce side effects.

In recent decades, various herb‐derived natural products have been reported to exhibit antioxidant or anti‐inflammatory effects.^6^ For example, Puerarin has been reported to have an anti‐inflammatory effect in RIR injury by inhibiting the activation of TLR4/NLRP3 inflammasome.^7^ However, in addition to the inflammation, the mechanisms of RIR injury also include oxidative stress and apoptosis. Therefore, the application of natural products with antioxidant and anti‐inflammatory effects in alleviating RIR injury has more important potential clinical significance. Quercetin (3,3′,4′,5,7‐pentahydroxyflavonoid; Que) is a natural flavonoid product found in many vegetables and fruits.^8^, ^9^ Que has a range of pharmacological effects including anti‐cancer,^10^ anti‐oxidant,^11^ and anti‐inflammatory^12^ effects. It has been reported to treat ischemia–reperfusion injury in multiple organs including the liver,^13^ kidney,^14^ heart,^15^ and cerebrum.^16^ Arikan et al. reported that intraperitoneal injection of Que dissolved in dimethyl sulfoxide (DMSO) attenuated the retinal thinning caused by RIR injury.^17^ As mentioned earlier, the eye is sensitive and has many barriers that reduce the efficiency of intraperitoneal injection, and the hydrophobic nature of Que makes vitreous injection difficult.

The rapid development of nanomaterials science has resulted in the emergence of many nanomedicines for intravitreal injection, which can deliver the hydrophobic compounds and bypass the barriers.^18^ Liposomes have shown promise as nanocarriers for the treatment of ocular diseases due to their biocompatibility, biodegradability, low toxicity, and efficient encapsulation of hydrophobic drugs.^19^ Although liposome‐based drugs for intravitreal injection have not yet been marketed, they have been reported to improve the intravitreal administration of drugs (e.g., vincristine).^18^ Some nanomicelles and inorganic nanoparticles have also been reported for use in ocular diseases, these nanomaterials have more disadvantages compared with liposomes. For example, poly (lactic‐co‐glycolic acid) nanomicelles have problems such as relatively low drug loading, inappropriate release rate, and potential toxicity,^20^ while inorganic nanoparticles have been reported disadvantages of low solubility and toxicity concerns.^21^ Liposomes have numerous advantages as drug carriers, which can significantly increase the duration of drug treatment effects, as well as drug levels in the posterior segment of the eye.^22^ However, one of the potential disadvantages of conventional liposomes is the burst release of nonspecific cargo in vivo.^23^ In recent years, a variety of ROS‐responsive materials have been synthesized and studied, including thiols, thioketals, hydroquinones, metallocenes, polypyridine ruthenium complexes, and thioethers.^24^ Increasing studies reported that ROS‐based nanomaterials can be used to treat myocardial ischemia–reperfusion injury.^25^, ^26^ In our previous study, we reported ROS‐responsive lipids (Di‐S‐PC) composed of thioether phosphatidylcholines in which the fatty acid chain of a typical lipid is replaced by two tails with thioether linkages.^27^ This ROS‐responsive element can be applied in stimuli‐responsive liposomes with good biocompatibility to obtain drug carriers targeting environments of oxidative stress. Thus, the design of ROS‐responsive liposomes would specifically improve the efficacy of the drugs.

Emerging evidences suggested that a major early event in RIR injury is ROS‐induced oxidative stress in the retina, which leads to retinal ganglion cell (RGC) loss, inflammation, and vascular dysfunction in posterior segment disorders.^28^, ^29^ Mitochondria are an important source of ROS in most mammalian cells.^30^ Therefore, we can construct mitochondria‐targeted nanomedicines to reduce ROS levels at earlier pathological stages. Triphenylphosphonium (TPP) is a positively charged lipophilic cation that preferentially accumulates in negatively charged mitochondria. Based on this feature, TPP has been conjugated on the surfaces of nanocarriers to develop a general mitochondria‐targeted drug delivery system. Recently, TPP‐based mitochondrial antioxidant delivery systems such as Mito‐TEMPO,^31^ MitoQ,^32^ MitoC,^33^ MitoE,^34^ TPP‐IOA,^35^ and lipid‐polymer hybrid nanoparticles^36^ have shown cardioprotective and antitumor effects by reducing mitochondrial ROS accumulation. TPP‐conjugated niacin protects against hydrogen peroxide (H_2_O_2_)‐induced cytotoxicity and mitochondrial dysfunction by upregulating antioxidant‐related genes in retinal pigment epithelial (RPE) cells.^37^ However, no in vitro or in vivo studies have been reported on the development of mitochondria‐targeted TPP‐conjugated liposome drugs for the treatment of ocular diseases.

**SCHEME 1:**
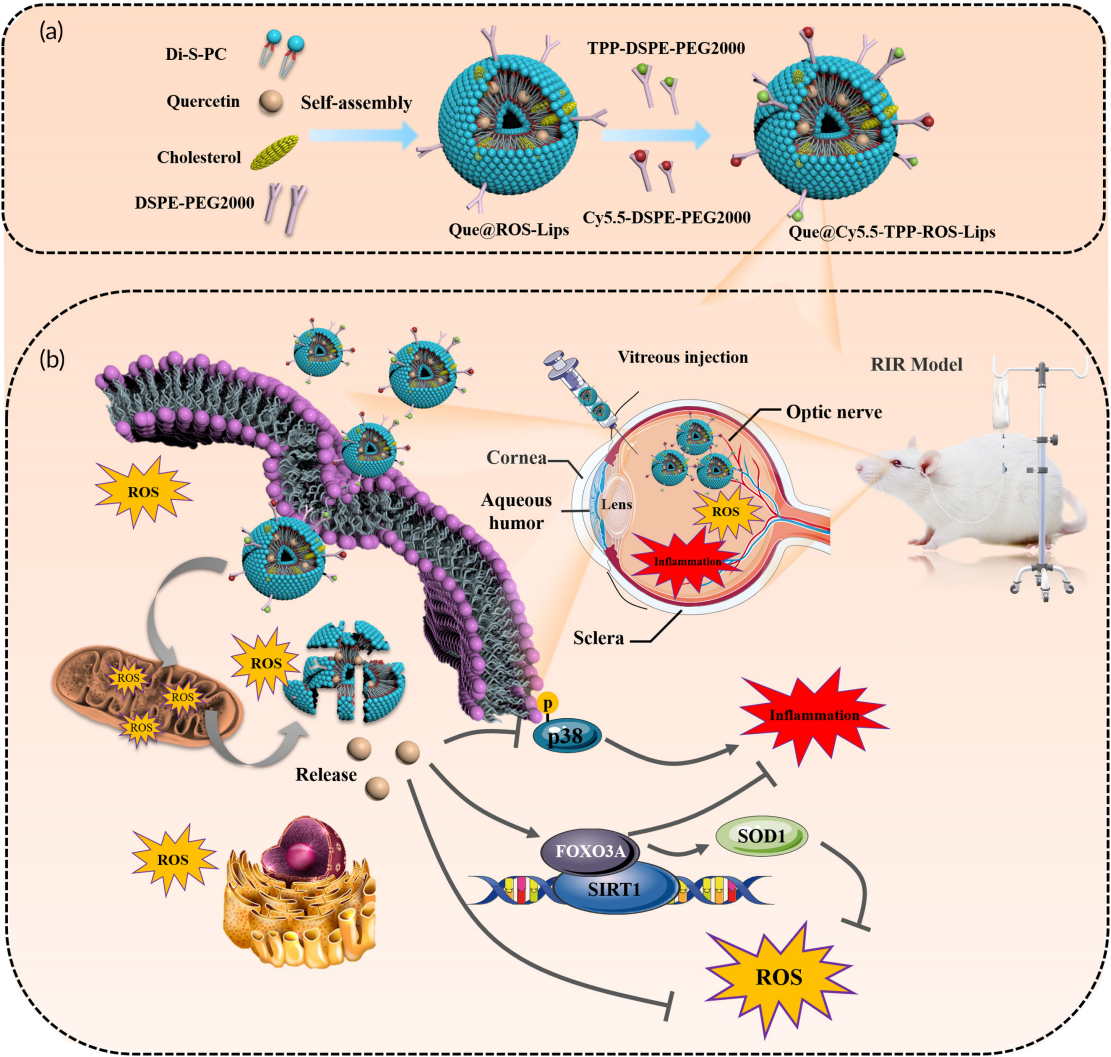
(a) Schematic of the preparation of Que@TPP‐ROS‐Lips for retinal ischemia therapy. (b) In vivo therapeutic mechanism of ROS‐responsive Lips in RGCs or glial cells based on the regulation of the SIRT1/FOXO3A and p38 pathways

To the best of our knowledge, no ROS‐responsive, mitochondria‐targeted lipid‐based nanosystem for the delivery of Que has been developed for the treatment of ocular diseases. In the present study, we developed a novel ROS‐responsive liposomal quercetin nanoformulation (Que@ROS‐Lips). The surfaces of the liposomes were functionalized by TPP to construct mitochondria‐targeted Que@ROS‐Lips (Que@TPP‐ROS‐Lips). We then evaluated the role of Que@TPP‐ROS‐Lips in RIR injury both in vitro and in vivo. The treatment of R28 cells with Que@TPP‐ROS‐Lips and the intravitreal injection of Que@TPP‐ROS‐Lips showed therapeutic effects in RIR injury in vitro and in vivo, respectively. Furthermore, mechanistic study showed that Que@TPP‐ROS‐Lips inhibited the activation of the mitogen‐activated protein kinase (MAPK) signaling pathway, which can promote oxidative stress and inflammatory response (Scheme 1). These findings provide a new strategy for the treatment of RIR injury and promote the clinical application of Que.

## 2 MATERIALS AND METHODS

### 2.1 Materials

Quercetin (Que) standard (purity ≥98%) was purchased from MedChemExpress (New Jersey, USA). Trichloromethane, methyl alcohol, and other chemicals were purchased from Sinopharm Chemical Reagent Co., Ltd. Soya bean lecithin (purity ≥98%) was purchased from Suzhou Meilun Biotechnology Co., Ltd. Cholesterol (purity ≥97%) was obtained from Accela ChemBio Co., Ltd. L‐α‐glycerophosphocholine (GPC; purity ≥98%), DSPE‐PEG2000, TPP‐DSPE‐PEG2000, FITC‐DSPE‐PEG2000, and Cy5.5‐DSPE‐PEG2000 were purchased from Xi'an Ruixi Biological Technology Co., Ltd (Shanxi, China). Dulbecco's modified eagle medium (DMEM), penicillin/streptomycin, and fetal bovine serum were obtained from Life Technologies (Carlsbad, CA, USA). We purchased 2′,7′‐dichlorofluorescin diacetate (H_2_DCF‐DA), dihydroethidium (DHE), neuronal Class III β‐TUBULIN (Tuj1) monoclonal antibody, GAPDH rabbit monoclonal antibody, IL‐1β rabbit polyclonal antibody, and IBA1 rabbit polyclonal antibody from Beyotime Biotechnology Co., Ltd. (Shanghai, China). Further, 4′,6‐diamidino‐2‐phenylindole (DAPI) was purchased from Amy Jet Scientific Inc Co., Ltd. (Wuhan, China).

### 2.2 Synthesis of ROS‐responsive lipids

A two‐step procedure similar to previously studies^27^, ^38^ was used to construct ROS‐responsive lipids (Di‐S‐PC). Briefly, to a solution of thioglycolic acid (0.56 g, 6.01 mmol) in 25% KOH solution in methanol, octadecane bromide (2 g, 6.01 mmol) was added and stirred vigorously at room temperature for 48 h. After acidification to pH 1 with aqueous HCl (0.1 M), the solution was extracted with ethyl acetate, dried over anhydrous NaSO_4_, and purified by column chromatography on silica gel (hexane/EtOAc, 3:1) to yield 0.86 g C18—S—COOH as a white solid (41% yield). ^1^H NMR (400 MHz, CDCl_3_) δ 3.25 (s, 2H), 2.69–2.63 (m, 2H), 1.61 (p, *J* = 7.3 Hz, 2H), 1.41–1.34 (m, 2H), 1.26 (s, 28H), 0.88 (t, *J* = 7.2 Hz, 3H).^13^C NMR (101 MHz, CDCl_3_) δ 175.43, 77.48, 77.16, 76.84, 33.63, 33.00, 32.08, 29.85, 29.74, 29.65, 29.51, 29.33, 29.07, 28.89, 22.84, 14.27, 1.17.

To a solution of C18—S—COOH (1 g, 2.91 mmol) in 15 ml of dichloromethane, *N*,*N*‐carbonyl diimidazole (0.71 g, 4.36 mmol) was added. Meanwhile, 1,8‐diazabicyclo[5.4.0]undec‐7‐ene (0.29 g, 1.94 mmol) and GPC (0.3 g, 1.16 mmol) were mixed in 15 ml of DMSO. After stirring for 1 h at 30°C, the first solution was transferred to the second for a further 24 h reaction. Without any postprocessing, the crude product was purified by silica column chromatography with gradient elution (A: CH_2_Cl_2_:CH_3_OH 5:1; B: CH_2_Cl_2_: CH_3_OH: H_2_O 65:25:4) to obtain 0.43 g (42.8% yield) of Di‐S‐PC lipid as a light‐yellow solid. The lipid structure was confirmed by ^1^H NMR, and ^13^C NMR (Figure S1B). ^1^H NMR (500 MHz, CDCl_3_) δ 5.26–5.23 (m, 1H), 4.43 (dd, *J* = 12.2, 3.0 Hz, 1H), 4.34–4.20 (m, 3H), 3.98–3.96 (m, 2H), 3.72–3.71 (m, 2H), 3.30–3.24 (m, 13H), 2.62–2.58 (m, 4H), 1.57 (p, *J* = 7.4 Hz, 4H), 1.38–1.35 (m, 4H), 1.26 (s, 56H), 0.88 (t, *J* = 6.8 Hz, 6H). ^13^C NMR (126 MHz, CDCl_3_) δ 170.57, 170.32, 71.63, 71.57, 66.42, 66.37, 63.80, 63.66, 63.44, 63.39, 59.53, 59.49, 54.47, 33.72, 33.58, 32.89, 32.81, 32.07, 29.90, 29.87, 29.82, 29.51, 29.17, 29.03, 28.99, 22.82, 14.25.

### 2.3 Preparation and characterization of liposomes

Que@ROS‐Lips and other liposomes were formulated using a thin‐film hydration method.^39^ Briefly, known quantities of lipids composed of Di‐S‐PC, lecithin, cholesterol, DSPE‐PEG2000, and Que (initial lipid/drug molar ratio = 30:1) were accurately weighed and dissolved in a chloroform/methanol (3:1, v/v) mixed solution for subsequent evaporation. The liposome particle size was homogenized by ultrasonication with 12 cycles of 15‐s sonication for 30 min (Thermo Fisher Scientific, Massachusetts, USA). After disruption in excess methanol, the nonentrapped Que within the liposomes was removed by centrifugation (5000 rpm, 20 min) to obtain the desired Que@ROS‐Lips. Que@TPP‐ROS‐Lips were prepared through a post‐procedure that involved adding 8% TPP‐DSPE‐PEG2000 (relative to total lipids) to Que@ROS‐Lips and incubating at 37°C for 2 h. Que@Cy5.5‐TPP‐ROS‐Lips and Que@FITC‐TPP‐ROS‐Lips were produced by the addition of Cy5.5‐DSPE‐PEG2000 or FITC‐DSPE‐PEG2000 (0.5% mol of total lipids) via a postinsertion method.

The particle hydrodynamic diameter, polydispersity index (PDI), and Zeta potentials of the liposomes were determined using a Delsa Nano C Particle Analyzer. The liposome morphology was observed by transmission electron microscopy (TEM). The Que in the liposomes was analyzed by high‐performance liquid chromatography (HPLC, Ultimate 3000; Thermo Fisher Scientific). Que solution with a certain concentration gradient was prepared with methyl alcohol, and the standard curve of Que concentration was constructed by HPLC. After centrifugation and filtration through a 0.22‐μm microporous filter, the liposomes were diluted and demulsified with methanol at 1:20 (v:v). The HPLC conditions were as follows: reverse C18 column (75 mm × 4.6 mm, 3.5 μm, Agilent); mobile phase, 65:35 methanol: water (v:v); injection volume, 10 μl; flow rate, 1.0 ml/min; and detection wavelength, 372 nm. The concentration of Que in the liposomes was then determined using the standard curve. The drug encapsulation efficiency (DEE) and drug loading content (DLC) of Que in the liposomes were, respectively, calculated as follows: DEE (%) = encapsulated Que amount/total Que amount × 100% and DLC (%) = encapsulated Que amount/total lipid amount × 100%.

### 2.4 In vitro investigation of ROS‐responsive drug release

The release of Que from liposomes in phosphate‐buffered saline (PBS; pH = 7.4) containing H_2_O_2_ (1.0 or 2.5 mM) was evaluated using a dialysis method.^40^ Briefly, 1.0 ml of Que@TPP‐ROS‐Lips was transferred into a cellulose ester dialysis bag (MWCO 1000 Da) and dialyzed against 40 ml of released medium under mechanical agitation at 150 rpm and 37°C in a platform shaker. At regular intervals, 4.0 ml of the supernatant was removed and replaced with an equivalent volume of fresh PBS or PBS containing different concentrations of H_2_O_2_ (pH = 7.4). The Que concentration in the released medium was determined by HPLC. The in vitro colloidal stability was determined by measuring the average particle size of Que@TPP‐ROS‐Lips over 14 days.

### 2.5 Cell culture

Retinal precursor R28 cells were obtained from Kerafast (Boston, MA, USA) and cultivated according to the supplier's instructions. The cells were grown in DMEM+, which included 420 ml DMEM (Sigma‐Aldrich, St. Louis, MO, USA), 15 ml sodium bicarbonate (Sigma‐Aldrich), 50 ml calf serum (HyClone), 5 ml MEM nonessential amino acids (GIBCO), 5 ml l‐glutamine (GIBCO), and 0.625 ml Gentamicin (Solarbio, Beijing, China). The cells were kept at 37°C in a humidified environment containing 5% CO_2_.

### 2.6 In vitro cellular uptake

R28 cells were cultured in a six‐well plate at a density of 1 × 10^5^/well to investigate the distribution of Que@TPP‐ROS‐Lips in the cells. After 24 h of culturing, the cells were incubated with fluorescently labeled liposomes. The final Que concentrations in the liposomes were 0, 5, 10, 20, 40, and 80 μM. Cells were collected after treatment for 24 h and rinsed twice with PBS, which removed nonintracellular LysoTracker dye. The R28 cells were detected by flow cytometer (CytoFLEX, Beckman Coulter).

### 2.7 Lysosome escape and mitochondria‐targeting ability

After nanoparticles enter cells, their localization within the cells is closely related to their ability to escape the lysosomes.^41^ Thus, we evaluated the ability of Que@TPP‐ROS‐Lips to escape lysosomes by confocal laser scanning microscopy (CLSM). The cells were incubated with fluorescent‐labeled liposomes (Lyso‐Tracker red) for various times (2, 4, 6, 8, and 16 h). After 30 min, the experiment was terminated by rinsing the cells three times with PBS, which removed the Lyso‐Tracker dye. The cells were then fixed with paraformaldehyde (4%, v/v), and the nuclei were stained with DAPI.

### 2.8 In vitro oxygen–glucose deprivation model and detection of cell activity

An in vitro oxygen–glucose deprivation (OGD) model was constructed based on the method detailed by Roth et al.^5^ Briefly, the cells were cultured in an anoxic, serum‐free environment for 24 h. The cells were then returned to normal culture conditions for 18 h to simulate RIR. The ATP levels were measured by CellTiter‐Lumi™ Plus luminescent cell viability assay (Beyotime, Beijing, China). The cell membrane integrity of the R28 cells was evaluated by lactate dehydrogenase (LDH) release assay (Beyotime).

### 2.9 Analysis of ROS content

The ROS in treated R28 cells were analyzed in vitro using an ROS assay kit (Beyotime). Briefly, the cells were incubated with liposomes for 24 h. OGD‐injured cells were incubated with H_2_DCF‐DA (10 μM) for 30 min at 37°C in the dark. Next, the cells were collected and washed with PBS three times, and cells were diluted with 500 μl of binding buffer and analyzed on a CytoFLEX flow cytometer (Beckman Coulter). The in vivo levels of retinal ROS were evaluated by staining 8‐μm‐thick retinal cryosections with DHE. The cryosections were fixed for 60 min at room temperature with 4% paraformaldehyde and then incubated in 10 μM of DHE for 30 min in the dark. After rinsing with PBS three times, the sections were counterstained and sealed with DAPI for observation by CLSM.

### 2.10 Measurement of glutathione level

The intracellular reduced glutathione (GSH) levels were assessed after the 24‐h treatment of OGD‐injured R28 cells with Que@TPP‐ROS‐Lips using GSH and GSSG Assay Kit (Beyotime) according to the manufacturer's suggestion.

### 2.11 Evaluation of mitochondrial membrane potential

Mitochondrial membrane potential (ΔΨm) is an important indicator of the physiological status of mitochondria. In this study, ΔΨm was evaluated using 5,5′,6,6′‐tetrachloro‐1,1′,3,3′‐tetraethylbenzimidazolyl‐carbocyanine iodide (JC‐1, Beyotime). Following treatment, cells were incubated with JC‐1 working solution for 20 min. Then the cells were washed three times with PBS and resuspended in binding buffer and analyzed on a CytoFLEX flow cytometer (Beckman Coulter). When the cells were in the normal state (high ΔΨm), red fluorescence appeared while green fluorescence indicated mitochondrial damage and decreased ΔΨm.

### 2.12 Animals

Eight‐week‐old male Sprague–Dawley rats (200–250 g) were purchased from Pengyue Experimental Animal Company (Jinan, China). The Yantai University Committee for the Care and Use of Laboratory Animals authorized the animal protocols, which followed the National Institutes of Health Guide for the Care and Use of Laboratory Animals in Research. All rats were housed under a 12‐h dark/light cycle and given unlimited access to water and food. The rats were anesthetized with intraperitoneal injections of ketamine (100 mg/kg) and xylazine (7 mg/kg) prior to intravitreal injection.

### 2.13 RIR injury model and intravitreal injection

RIR injury was established in rats using a previously published procedure.^29^ The conjunctival sac of each anesthetized rat was cleaned with 0.3% ofloxacin eye drops. Tropicamide eye fluid was used to dilate the pupils, proparacaine hydrochloride was used as a local anesthetic, and tobramycin was used to prevent infection after surgery. We inserted a 30‐gauge, 5/8‐inch metal needle (BD Precision Glide, Becton‐Dickinson, Franklin Lakes, NJ) from the temporal corneal limbus into the anterior chamber of the left eye and connected to a sterile saline reservoir. The infusion device was monitored for successful infusion while the needle was oriented toward the optic disk and fastened with a conjunctival suture and adhesive tape. The lens and cornea were shielded during this process. The infusion bottle was lifted to a vertical distance of 150 cm, and the intraocular pressure (IOP) of the rats was found to be approximately 110 mmHg using a pressure flush tonometer. Ophthalmoscopic examination revealed pallor of the conjunctiva and iris, lightening of the retina, and extensive occlusion of central retinal arteriovenous blood flow. After 60 min, the needle was removed for normalization of IOP and reperfusion of retinal vessels, which was confirmed by ophthalmoscopy. Posterior segment pallor and recovery of blood supply confirms successful establishment of RIR injury model. To avoid infection, erythromycin eye ointment was administered to both eyes after the puncture needle was removed. Each animal's body temperature was kept between 36.5 and 37°C during the experiment, and the corneas were wetted with 0.3% ofloxacin eye drops.

At 24 h after the RIR model was established, the RIR injury rats were randomly divided into six groups: saline, TPP‐ROS‐Lips, Que@Lips, Que@ROS‐Lips, Que@TPP‐Lips, and Que@TPP‐ROS‐Lips. The methods for anesthesia and pupil dilation were the same as those described above. Intravitreal injection was accomplished using a Hamilton syringe; 4 μl of Que@TPP‐ROS‐Lips was injected to obtain a final Que concentration of 20 μM in the vitreous humor.^42^

### 2.14 Preparation of frozen retina sections and immunofluorescent staining

The model rats were euthanized at 7 days after the intravitreal injection of liposomes. Immediately after the eyeball was removed, it was fixed in 4% paraformaldehyde for 2 h at 4°C, dehydrated overnight with 30% sucrose solution, embedded in an optimal cutting temperature compound (OCT) embedding bottom box (17 × 17 × 5 mm), and stored at −80°C. Slices with thicknesses of 8 μm were created using a frozen slicer at −22°C. The slices were placed on adhesive slides and dried at room temperature for more than 30 min before proceeding to the next step. For immunofluorescent staining, the retinal sections were permeabilized with enhanced immunostaining permeabilization buffer (Beyotime) for 20 min, blocked with blocking buffer (Beyotime), and incubated overnight at 4°C with primary antibodies: Class III β‐TUBULIN (Tuj1 mAb) (Beyotime), Rabbit anti‐IBA1/AIF‐1, rabbit anti‐FOXO3A and rabbit anti‐SIRT1(Cell Signaling Technology). After rinsing three times with PBS, the sections were incubated with secondary antibodies at room temperature for 2 h, rinsed three times with PBS, and counterstained with DAPI for 5 min. The slides were then sealed with cover glass for observation by CLSM.

### 2.15 Evaluation of retinal function

All electroretinography (ERG) procedures were performed in the dark room under dim red‐light illumination (>650 nm).^43^ ERG recordings were conducted for four groups: sham, saline, TPP‐ROS‐Lips, and Que@TPP‐ROS‐Lips. The rats were dark‐adapted overnight before receiving intraperitoneal 1% pentobarbital (3 ml/kg) and 50% isoflurane (250 μl/kg) anesthesia. Tropicamide eye fluid was used to dilate the pupils under dim red‐light illumination. Following anesthesia and mydriasis, stainless‐steel subdermal needle electrodes were implanted as the ground (at the tail) and reference (beneath individual eyelids) electrodes. The cornea was kept moist by applying 0.1% sodium hyaluronate eye drops (Santen Pharmaceutical) to the recording gold electrodes. The animals were placed on a thermal platform that was kept at 37°C. Under dark‐adapted conditions, the intensity of white light stimulation for scotopic ERG was initially adjusted to 0.01 cd∙s∙m^−2^, and flash ERG recordings were obtained concurrently from both eyes. The intensity was then increased to 3.0 cd∙s∙m^−2^. At the above luminance levels, each recording was averaged three times.

### 2.16 Evaluation of mitochondria morphology in the retina

Following retina dissection, the tissues were immediately fixed in 2.5% phosphate glutaraldehyde for 2 h at 4°C. The samples were then post‐fixed, embedded, cut, and mounted at Zhejiang University's Electron Microscopy Core Facility. All samples were examined by TEM at an accelerating voltage of 100 kV.^3^

### 2.17 Western blot analysis

Total cellular proteins were extracted from OGD‐injured R28 cells, and the protein concentrations were determined using a BCA protein assay kit (Beyotime). The primary antibodies used were FOXO3A, SIRT1, p38, and phospho‐p38 MAPK (Cell Signaling Technology, Danvers, MA, USA). Glyceraldehyde‐3‐phosphate dehydrogenase (GAPDH, Proteintech, Wuhan, China) served as the loading control for total proteins. For each treatment, three independent experiments were conducted for western blot analysis. The intensities of the resulting bands were quantified using ImageJ software (Version 1.52p, NIH).

### 2.18 Cytokine measurement by ELISA

Retinal tissue was homogenized with a low‐temperature homogenizer (ServiceBio, Wuhan, China) and supernatants were then collected for measurement of IL‐1β and TNF‐ α by ELISA Kits (Beyotime) following the manufacturer's instructions.

### 2.19 Safety evaluation

In the section of normal rats, normal rats were intravitreal injection of Que@TPP‐ROS‐Lips. The rats were euthanized after 7 or 14 days, and the eyeballs were harvested for hematoxylin and eosin (H&E) staining to observe retinal changes. Frozen sections were used to detect the protein expression levels of β‐III‐TUBULIN and BRN3A in the retina 7 days after administration. ERG was used to detect the safety of the retinal function after 7 days of administration. In the section of Safety assessment for pathological conditions with RIR impairment, orbital blood was taken to detect the contents of alanine transferase and aspartic transferase in plasma. The heart, liver, spleen, lung, kidney, and other major organs were harvested for H&E staining to observe changes in each major organ.

### 2.20 Statistical analysis

All data are presented as the mean ± standard deviation (SD). Statistical analyses were performed using GraphPad Prism 6 (La Jolla, USA). Treatment‐related differences were evaluated by one‐way analysis of variance (ANOVA) followed by Dunnett's test (for comparisons between different concentrations and the vehicle control) or two‐way ANOVA followed by Tukey's multiple comparison test (for comparisons between different treatment groups). A difference was considered statistically significant if the *p* value was less than 0.05.

## 3 RESULTS AND DISCUSSION

### 3.1 Preparation and characterization of Que‐loaded liposomes

**FIGURE 1:**
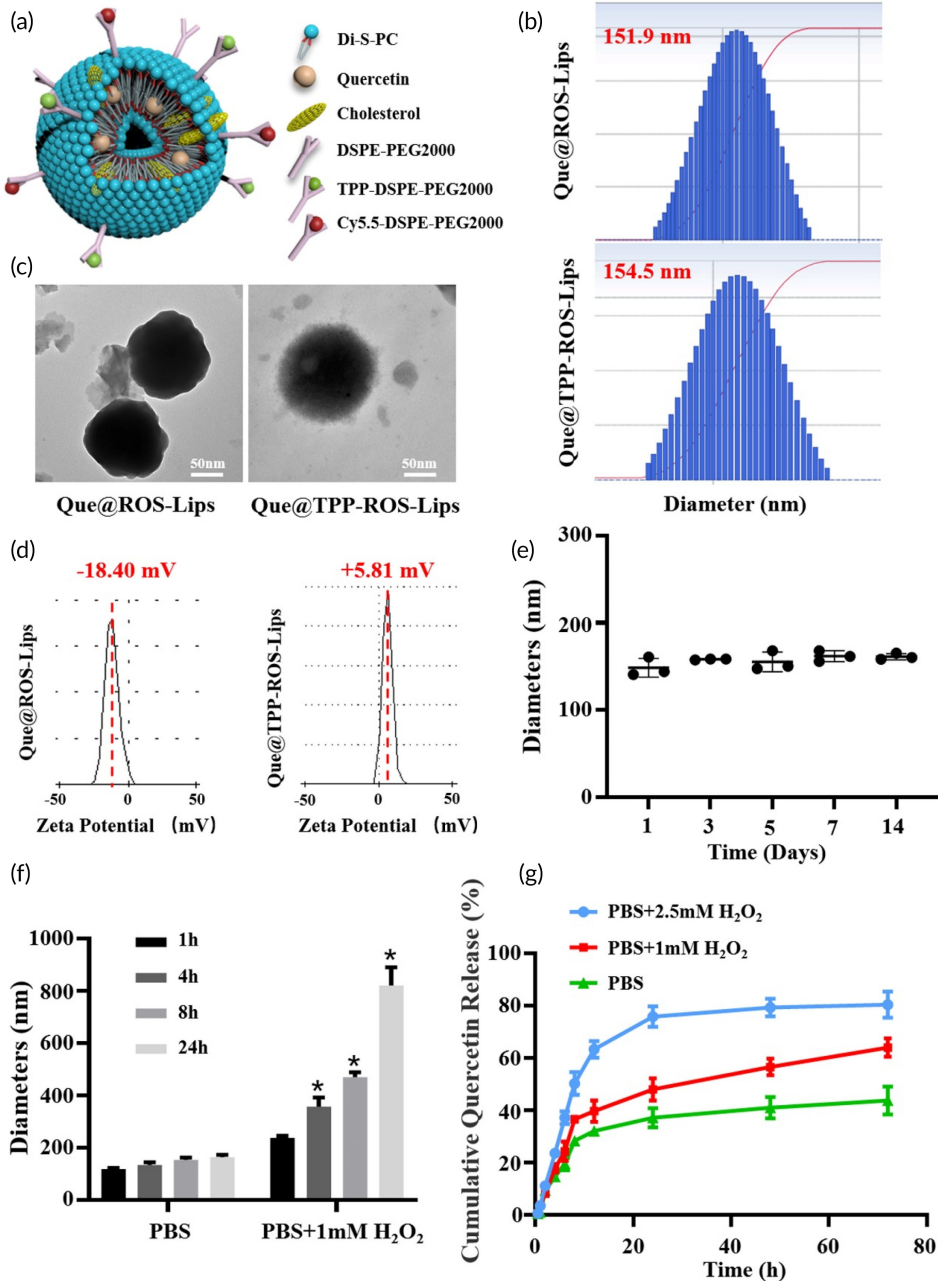
Characterization and release profiles of Que@TPP‐ROS‐Lips. (a) Three‐dimensional schematic illustration of Que@TPP‐ROS‐Lips. (b) Size distributions of Que@ROS‐Lips and Que@TPP‐ROS‐Lips determined by dynamic light scattering (DLS). (c) transmission electron microscopy (TEM) images of Que@ROS‐Lips and Que@TPP‐ROS‐Lips (scale bar = 50 nm). (d) Zeta potential of Que@ROS‐Lips and Que@TPP‐ROS‐Lips determined by DLS. (e) Stability of Que@TPP‐ROS‐Lips diameter over 14 days (*n* = 3). (f) Change in diameter under stimulated physiological (PBS) and pathological (PBS with 1.0 mM H_2_O_2_) conditions (*n* = 3). (g) Release profiles of Que@TPP‐ROS‐Lips in the presence of 0, 1, and 2.5 mM H_2_O_2_. Data are presented as mean ± SD (*n* = 3).

In the present study, we conjugated liposomes with Di‐S‐PC lipids for ROS response. In addition, TPP and Cy5.5 dye were used to conjugate in the liposomes for targeting mitochondria and Lip‐tracing, respectively. A schematic of Que@TPP‐ROS‐Lips is shown in Figure 1a. Di‐S‐PC lipids structures were determined by ^1^H NMR and ^13^C NMR (Figure S1). Que@ROS‐Lips were prepared by thin‐film hydration, and lecithin and DSPE‐PEG2000 were added to increase the liposome stability. After the preparation of Que@ROS‐Lips, TPP‐DSPE‐PEG2000 and Cy5.5‐DSPE‐PEG2000 (or FITC‐DSPE‐PEG2000) were added to obtain the targeted Que@TPP‐ROS‐Lips (Scheme 1). The average hydrodynamic diameter of Que@ROS‐Lips was similar to that of Que@TPP‐ROS‐Lips and both showed uniform particle‐size distributions (Figure 1b). The appearance of Que@ROS‐Lips (151.9 nm) did not differ appreciably compared with Que@TPP‐ROS‐Lips (154.5 nm); however, TEM revealed a change in surface morphology from smooth to rough after TPP modification (Que@TPP‐ROS‐Lips; Figure 1c). The zeta potential of the liposomes increased from −18.4 to +5.76 mV after the addition of TPP (Figure 1d), indicating that a large number of TPP molecules were chemically conjugated to the surface of liposomes. Dynamic light scattering (DLS) measurements at 37°C for various time intervals demonstrated the physiological stability of Que@TPP‐ROS‐Lips, which retained a constant particle size (Figure 1e) over 14 days. DLS also confirmed the ROS‐responsive feature of Que@TPP‐ROS‐Lips and Que@TPP‐Lips; Que@TPP‐ROS‐Lips swelled to approximately 5.3 times its initial size in response to 1 mM H_2_O_2_, whereas the size did not change significantly in PBS without H_2_O_2_ (Figure 1f). Additionally, the particle size of Que@TPP‐Lips was also assessed in PBS containing 1 mM H_2_O_2_. As shown in Figure S2A, the particle size of Que@TPP‐Lips has no time‐dependent change in PBS containing 1 mM H_2_O_2_, which was different from that of Que@ROS‐TPP‐Lips, suggesting Que@TPP‐Lips have no H_2_O_2_ responsiveness. This is consistent with the assumption that Que@TPP‐ROS‐Lips are disassembled via the oxidative destabilization of sulfide moieties to sulfoxides and then sulfones, which increases the hydrophilicity of the initially hydrophobic lipids.^44^ The efficiency of drug encapsulation is an important metric for drug delivery systems. The DEE and DLC of Que in the liposomes were 90.2% ± 4.67% and 4.30% ± 0.98%, respectively; modification with TPP had little effect on the Que loading in Que@TPP‐ROS‐Lips.

The Que release profiles from the lipids were then evaluated under simulated physiological (PBS, pH = 7.4) and pathological (PBS, pH = 7.4 with 1.0 and 2.5 mM H_2_O_2_) conditions. As shown in Figure 1g, Que@TPP‐ROS‐Lips remained stable with a low Que release rate (40%) in PBS after 72 h, and the Que release rate showed time‐dependent increase in PBS containing 1.0 mM H_2_O_2_. Que@TPP‐ROS‐Lips showed the highest Que release rate in PBS containing 2.5 mM H_2_O_2_, as evidenced by the 80% release rate of Que at 72 h. In contrast, Que@TPP‐Lips did not show significant difference in Que release rate between PBS and PBS containing different concentrations of H_2_O_2_ (Figure S2B). This can be explained by the sensitivity of the Di‐S‐PC lipids to high concentrations of H_2_O_2_, which contributes to the collapse of the liposome nanostructure and drug release.^45^ Large amounts of ROS were produced in the retina during RIR; thus, the ROS‐responsive behavior of Que@TPP‐ROS‐Lips would lead to the release of Que after RIR injury, resulting in a good therapeutic effect. These findings suggest that Que@TPP‐ROS‐Lips have excellent potential for clinical application in the treatment of oxidative damage‐related diseases. To the best of our knowledge, the construction and application of ROS‐responsive Que liposomes have not previously been reported. Therefore, we generated a novel liposomal drug delivery nanoplatform tailored for targeting and protection of retinal cells damaged by oxidative stress.

### 3.2 Intracellular uptake, lysosome escape ability, and mitochondria targeting ability of Que@TPP‐ROS‐Lips

**FIGURE 2:**
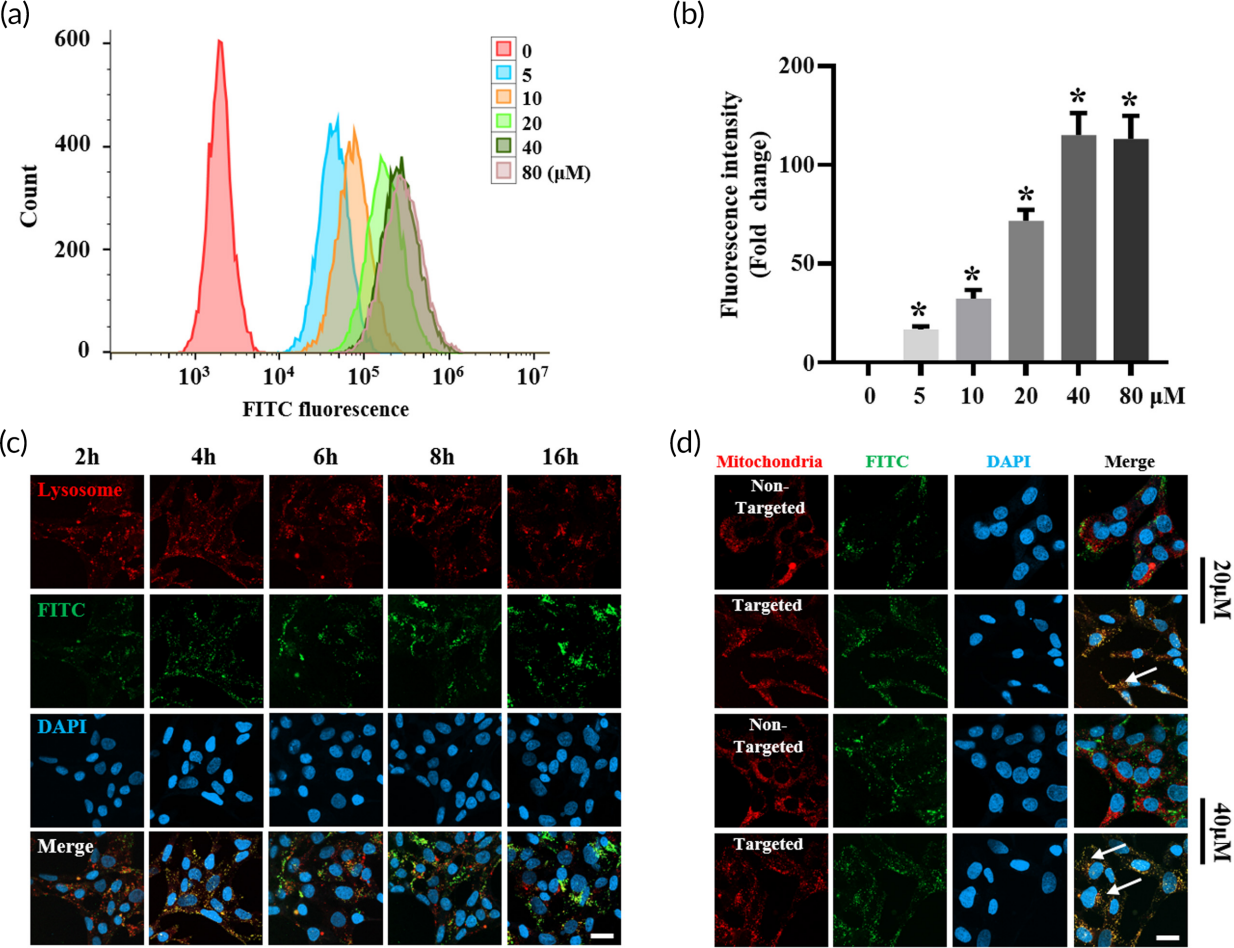
In vitro intracellular uptake and mitochondrial targeting ability. (a, b) Concentration‐dependent cellular uptake of Que@FITC‐TPP‐ROS‐Lips in R28 cells after 24 h of incubation. FITC fluorescence is from Que@FITC‐TPP‐ROS‐Lips. Representative flow cytometry data are shown in panel (a). Quantification of FITC fluorescence is shown in panel (b). *p < 0.05 (c) Lysosome escape ability of Que@ FITC‐TPP‐ROS‐Lips after 2, 4, 6, 8, and 16 h. Orange fluorescence (the combination of red and green fluorescence) indicates Que@FITC‐TPP‐ROS‐Lips (green) accumulation in the lysosomes (red). (d) Intracellular localization of Que@FITC‐TPP‐ROS‐Lips and Que@FITC‐ROS‐Lips. The orange fluorescence (combination of red and green fluorescence) indicates the localization of Que@FITC‐TPP‐ROS‐Lips (green) in the mitochondria (red). Data are presented as mean ± SD (*n* = 3). Scale bars = 20 μm

Flow cytometry was performed to examine the effect of liposome uptake by R28 cells. As shown in Figure 2a, the data demonstrate the obvious internalization of Que@TPP‐ROS‐Lips modified by Fluorescein isothiocyanate (FITC). Figure 2b shows the quantitative analysis result of Que@TPP‐ROS‐Lips internalized in R28 cells. The endocytosis was enhanced in a concentration‐dependent manner and was maximized at a concentration of 40 μM Que@FITC‐TPP‐ROS‐Lips (Figure 2a,b). The ability to escape lysosomes is an important prerequisite for the nanomedicine to achieve subcellular localization. After Que@TPP‐ROS‐Lips entered into the cells, the lysosome escape ability was observed at different time points by CLSM. Lysosomes emitted red fluorescence based on Lyso‐tracker, and FITC‐labeled liposomes emitted green fluorescence (Figure 2c). Orange fluorescence (combination of red and green fluorescence) indicated that Que@TPP‐ROS‐Lips accumulated in the lysosomes. After 2 h of incubation, green liposome (FITC‐labeled liposomes) fluorescence was mainly localized in the lysosomes, showing orange fluorescence (Figure 2c). When the incubation time was extended to 4 h, strong green fluorescence was observed, indicating that Que@TPP‐ROS‐Lips effectively escaped the lysosomes. Interestingly, this escape ability was time dependent. Following 16 h of incubation, the FITC fluorescence intensity was at its strongest (Figure 2c). The ability of a nanomaterial to escape from lysosomes in cells is an important factor for practical applications.^46^, ^47^ In addition, the mitochondrial localization of Que@TPP‐ROS‐Lips in R28 cells was also evaluated by CLSM. FITC‐labeled Que@TPP‐ROS‐Lips emitted green fluorescence, Mito‐Tracker emitted red fluorescence in the mitochondria, and the orange fluorescence (the combination of green and red fluorescence) indicated the localization of Que@TPP‐ROS‐Lips in the mitochondria. As shown in Figure 2d, compared with the Que@ROS‐Lips group, stronger orange fluorescence was observed in the Que@TPP‐ROS‐Lips group, and no significant difference in FITC fluorescence intensity was detected between the Que@TPP‐ROS‐Lips and Que@ROS‐Lips liposomes after incubation. Thus, these results indicate that after escape from the lysosomes, Que@TPP‐ROS‐Lips successfully targeted the mitochondria. To date, only two studies have explored TPP‐conjugated drugs for the treatment of ocular oxidative stress: one reported a therapeutic effect in RPE cells, while the other focused on RPE and human cornea cells.^37^, ^48^ Moreover, to the best of our knowledge, there are no studies reporting the application of TPP‐conjugated nanomaterials in RIR injury.

### 3.3 Que@TPP‐ROS‐Lips attenuates decreased cell viability and oxidative stress in R28 cells subjected to OGD

**FIGURE 3:**
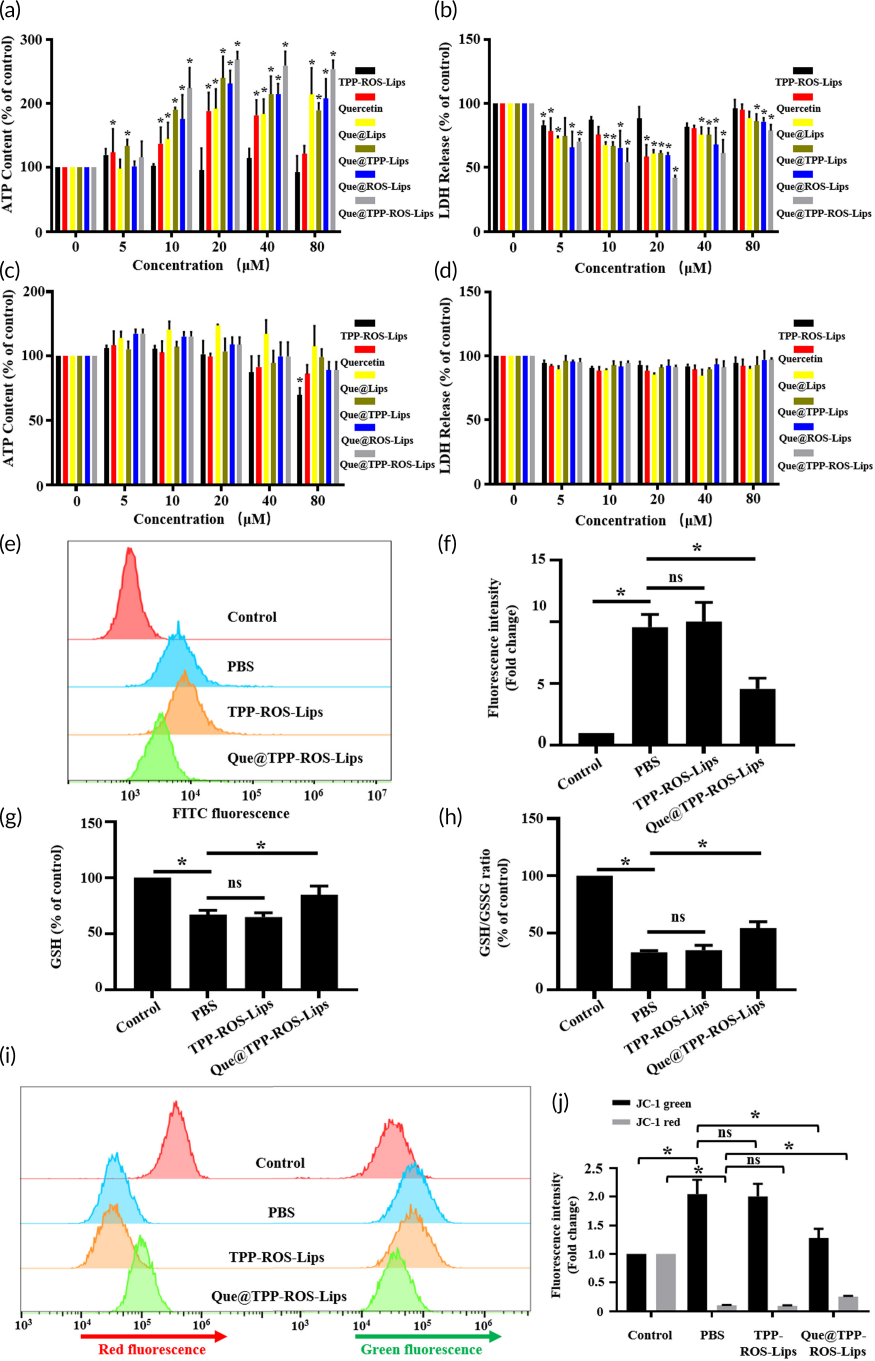
Que@TPP‐ROS‐Lips protected R28 cells subject to OGD. (a) Cellular ATP content and (b) LDH release showing the dose‐dependent effect of Que@TPP‐ROS‐Lips on OGD‐induced cytotoxicity in R28 cells. (c) TPP‐ROS‐Lips (80 μM) caused a slight decrease in the ATP level in R28 cells under normal conditions but showed no toxicity based on LDH release (d). ROS (e), GSH (g), GSH/GSSG (h), and ΔΨm (i) in R28 cells pretreated with PBS, TPP‐ROS‐Lips, and Que@TPP‐ROS‐Lips were determined using H_2_DCF‐DA, GSH Assay kit and JC‐1 dye, respectively (*n* = 6). Quantifications of fluorescence intensity of ROS and JC‐1 are shown in panels (i, j), respectively. Data are presented as mean ± SD (*n* = 6); *p < 0.05 compared to the PBS group.

In this study, the efficacy of Que@TPP‐ROS‐Lips in vitro was examined using the OGD model, a widely reported model for ischemia–reperfusion injury in multiple organs.^5^, ^49^, ^50^, ^51^ During the establishment of the OGD model, if the re‐oxygenate culture time after hypoxia is too long, the cells will be close to normal physiological conditions without any treatment (data not shown). Therefore, we pretreated the cells before establishing the OGD model as this mode of administration would ensure the duration of action of Que@TPP‐ROS‐Lips without making the re‐oxygenation time too long in vitro. Therefore, to investigate the role of Que@TPP‐ROS‐Lips in an in vitro OGD model, R28 cells pretreated with PBS for 24 h were subjected to OGD. As shown in Figure 3a,b, in the absence of Que@TPP‐ROS‐Lips, OGD induced cytotoxicity, as evidenced by significant reductions in cellular ATP content (Figure 3a) and increased LDH release (Figure 3b). Pretreatment with TPP‐ROS‐Lips, Que, Que@Lips, Que@TPP‐Lips, Que@ROS‐Lips, and Que@TPP‐ROS‐Lips significantly enhanced cell viability in a dose‐dependent manner, with Que@TPP‐ROS‐Lips resulting in the highest viability. For example, after pretreatment with 20 μM Que@TPP‐ROS‐Lips, the maximum ATP content was 2.68‐fold that of the PBS control (Figure 3a). Under normoxic conditions, no obvious changes were observed in ATP content (Figure 3c) or LDH release (Figure 3d) after pretreatment with TPP‐ROS‐Lips, Que, Que@Lips, Que@TPP‐Lips, Que@ROS‐Lips, and Que@TPP‐ROS‐Lips. Only the group of R28 cells treated with the highest concentration of TPP‐ROS‐Lips (equivalent amount of TPP‐ROS‐Lips that would have encapsulated 80 μM of Que) showed a decrease in ATP levels (Figure 3c); no toxicity was apparent from the LDH release results (Figure 3d). Thus, among the treatments, Que@TPP‐ROS‐Lips exhibited the best efficacy in the OGD model and did not exhibit obvious toxic effects.

Oxidative stress is an early event in retinal ischemia.^29^, ^52^ We tested the hypothesis that Que@TPP‐ROS‐Lips can reverse the oxidative stress caused by ischemic injury in vitro and in vivo. ROS accumulation is typical during oxidative stress.^53^, ^54^ Therefore, in the present study, R28 cells were pretreated with Que@TPP‐ROS‐Lips at concentrations of 20 μM for 24 h and subjected to OGD and then ROS production was monitored. Compared to the OGD/PBS group, pretreatment with Que@TPP‐ROS‐Lips significantly decreased the level of ROS‐induced by OGD in a concentration‐dependent manner (Figure 3e,f). Glutathione exists in cells in reduced (GSH) and oxidized (GSSG) states, and the harmful effects of ROS can be counteracted by GSH. Therefore, to evaluate the role of Que@TPP‐ROS‐Lips in OGD induced ROS, the levels of GSH and GSSG were measured at 24 h after treatment to calculate the GSH/GSSG ratios. As shown in Figure 3g,h, levels of GSH and the GSH/GSSG ratio significantly increased in the cells treated with 20 μM of Que@TPP‐ROS‐Lips. Since mitochondria are the main site of ROS generation, the accumulation of ROS is likely to cause mitochondrial dysfunction. Mitochondrial function is commonly evaluated based on ΔΨm, with a decrease in ΔΨm indicating mitochondrial depolarization and the loss of mitochondrial function.^54^ We examined the effect of Que@TPP‐ROS‐Lips on OGD‐related changes in ΔΨm using JC‐1 staining. As shown in Figure 3i,j, the transition from red fluorescence to green fluorescence in the JC‐1 staining images was more obvious in the Que@TPP‐ROS‐Lips group compared with the PBS group, suggesting that Que@TPP‐ROS‐Lips attenuated the OGD‐induced mitochondrial depolarization. Taken together, Que@TPP‐ROS‐Lips significantly inhibit OGD‐induced cytotoxicity and oxidative stress in R28 cells. This observation of anti‐oxidative stress activity of Que@TPP‐ROS‐Lips is congruent with a previous RIR injury study in which the role of SOD nanoformulations in RIR injury was attributed to their antioxidant effect.^29^ The findings of our study indicated that Que@TPP‐ROS‐Lips inhibit the early pathological process of RIR injury and this encouraged further evaluation of Que@TPP‐ROS‐Lips following ischemic injury in rodent models.

### 3.4 Que@TPP‐ROS‐Lips administration following retinal ischemia in vivo attenuates ischemic damage

**FIGURE 4:**
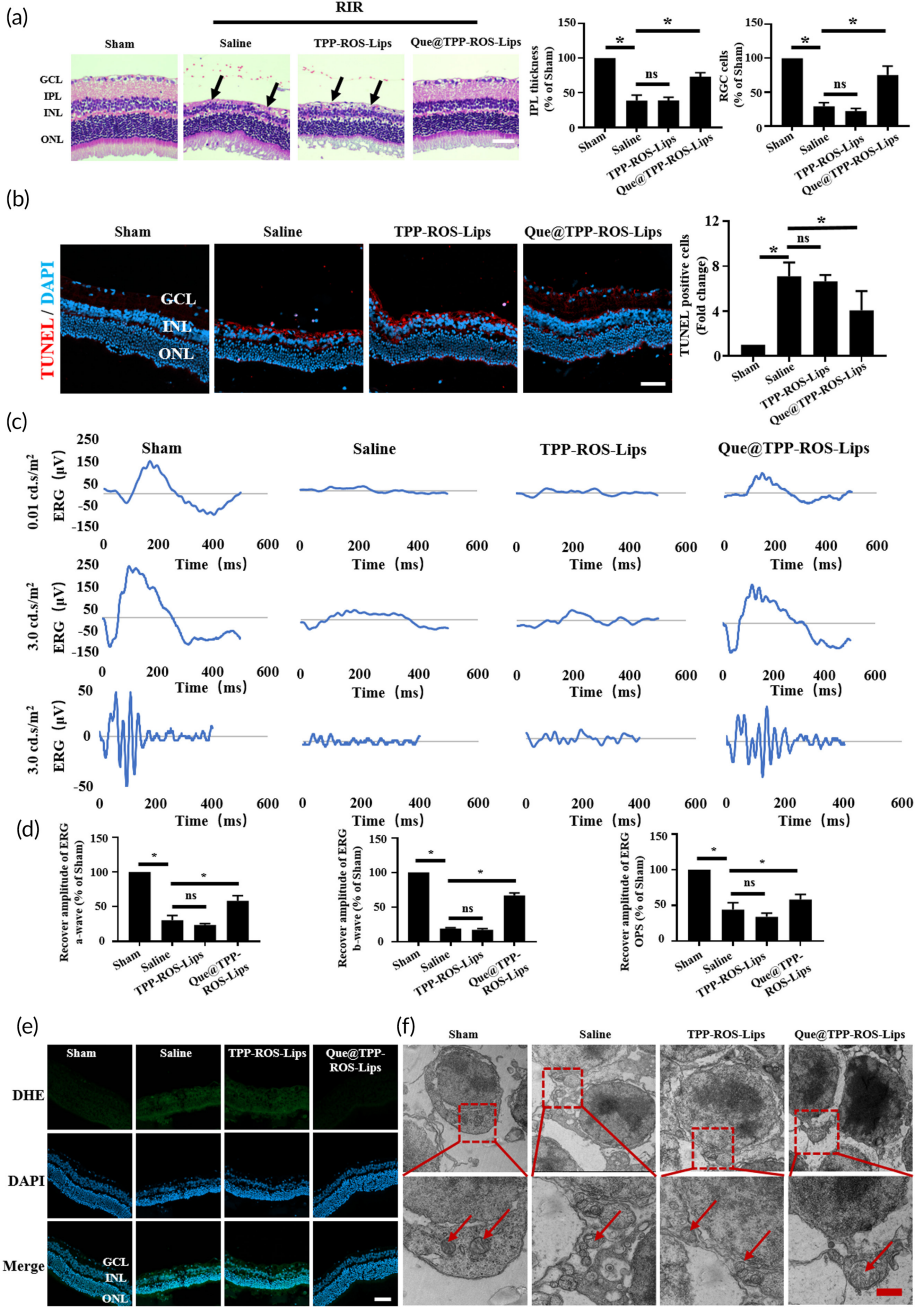
Intravitreal injection of Que@TPP‐ROS‐Lips protects RGCs and retinal function after RIR injury in vivo. (a) H&E staining images of retinal paraffin sections in different groups. The black arrows indicate RGCs. Scale bars = 20 μm; *n* = 6; GCL, ganglion cell layer; IPL, inner plexiform layer; INL, inner nuclear layer; ONL, outer nuclear layer. Retinas exposed to RIR injury showed significantly decreased IPL thickness (left) and increased RGC loss (right). (b) Representative TUNEL staining images (left panel of retinal cryosections suggesting that Que@TPP‐ROS‐Lips decreased in TUNEL positive cells in ischemic retinas compared to saline pretreated retinas. Quantification of TUNEL positive cells (right panel). (c) Representative ERG traces at 0.01 and 3.00 cd∙s∙m^−2^ from rats in the sham, saline, TPP‐ROS‐Lips, and Que@TPP‐ROS‐Lips groups. (d) Amplitudes of the scotopic ERG a (left) and b (middle) waves and sums of the OPs amplitudes (right) in the sham, saline, TPP‐ROS‐Lips, and Que@TPP‐ROS‐Lips groups at the light intensity of 3.00 cd∙s∙m^−2^. (e) Ability of Que@TPP‐ROS‐Lips to scavenge ROS in vivo based on DHE staining and immunocytochemistry (scale bar = 50 μm; *n* = 6). (f) TEM images showing the morphologies of mitochondria in retinal tissues from the sham, saline, TPP‐ROS‐Lips, and Que@TPP‐ROS‐Lips groups. Scale bars are 2 μm for the upper images and 1 μm for the lower images. Data are presented as mean ± SD (*n* = 6). *p < 0.05 compared with the sham group

To further explore the efficacy of Que@TPP‐ROS‐Lips in vivo, we conducted experiments in a rat model of RIR injury. Owing to the detection of various indicators 7 days after modeling, the intravitreal injection of Que@TPP‐ROS‐Lips was performed after the establishment of model, and this administration also mimics the drug delivery in clinical treatment. Moreover, in vitro and in vivo modes of administration in our study were also consistent with the previous study on the role of exosomes in RIR injury.^5^ In this in vivo model of RIR injury, Que@TPP‐ROS‐Lips were administrated by intravitreal injection, which is the clinically preferred administration for posterior segment eye diseases for several advantages: (1) the drug is administrated into the vitreous humor, which is in proximity to the retina; (2) the administration bypasses various barriers; and (3) systemic side effects are minimized.^4^ Although retinal delivery by intravitreal injection is reportedly hindered by the vitreous and vitreoretinal interface,^55^ it is by far remains the most effective clinical methods for treating posterior segment eye diseases.^56^ However, conventional injectable drugs require frequent intravitreal administration, which causes serious side effects in patients. The sustained‐release effect of Que@TPP‐ROS‐Lips constructed in this study can reduce the number of required injections. During RIR, RGCs are damaged, and the thickness of the inner plexiform layer (IPL) decreases, leading to the loss of RGCs.^52^, ^57^ In the current study, compared to normal retinas, retinas exposed to RIR for 7 days showed a significant reduction in IPL thickness. Moreover, the administration of Que@TPP‐ROS‐Lips significantly alleviated this decrease in thickness (Figure 4a). Consistent with these findings, the H&E staining images indicated that the loss of RGCs induced by RIR injury was significantly attenuated after Que@TPP‐ROS‐Lips administration (Figure 4a right panel). In addition, TUNEL staining indicated that Que@TPP‐ROS‐Lips administration significantly decreased RIR injury‐induced apoptosis in retinal cells (Figure 4b).

ERG, which is used to monitor retinal function and disease progression,^58^ was applied to assess the effect of Que@TPP‐ROS‐Lips on retinal function at 7 days after RIR injury. The ERG results were normalized to the baseline prior to ischemia and to control eyes. As shown in Figure 4c, significant improvements of recovery in the a and b waves were evident in representative ERG stimulation intensities. Compared to the saline control, intravitreal Que@TPP‐ROS‐Lips treatment significantly improved the recovery of the a‐ and b‐wave amplitudes (Figure 4c). OPs are considered to arise within the IPL due to an inhibitory feedback loop mainly involving amacrine cells.^58^ In the present study, OPs were also monitored. The sum of OPs amplitudes was significantly lower in the RIR/saline group compared to in the sham group, while that in the Que@TPP‐ROS‐Lips group was significantly higher than that in the RIR/saline group (Figure 4d).

Consistent with the in vitro results of ROS overproduction, the DHE staining images showed significantly higher ROS accumulation in the RIR/saline group compared with the sham group. The intravitreal injection of Que@TPP‐ROS‐Lips significantly decreased the level of ROS compared with the normal saline group (Figures 4e and S3). Additionally, we examined the morphologies of the mitochondria in retinal cells subject to RIR injury by TEM. The retinal mitochondria in the RIR/saline group were significantly enlarged and distorted compared with those in the sham group. The administration of Que@TPP‐ROS‐Lips partially reversed these effects (Figure 4f). These results demonstrate that Que@TPP‐ROS‐Lips attenuated the accumulation of ROS and the reduction in mitochondrial membrane potential caused by RIR injury. Taken together, these findings indicate that treatment with Que@TPP‐ROS‐Lips following RIR injury leads to functional improvement in the retina.

### 3.5 Que@TPP‐ROS‐Lips attenuate the loss of RGCs caused by RIR injury

**FIGURE 5:**
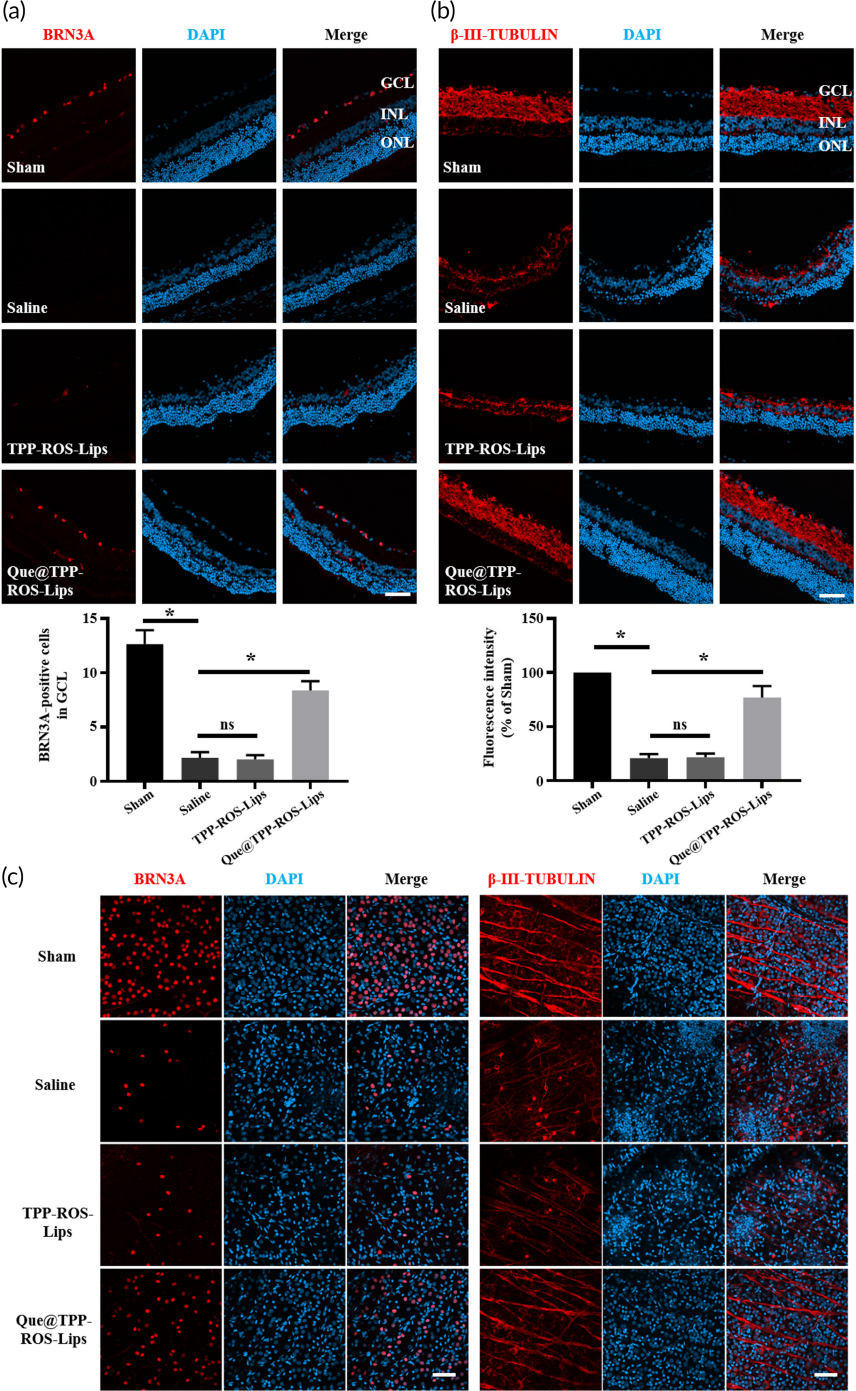
Intravitreal injection of Que@TPP‐ROS‐Lips reverses the damage to RGCs caused by RIR injury. Representative CLSM images and statistical results of fluorescence showing the levels of RGC markers, BRN3A (a) and β‐III‐TUBULIN (b), in retinal cryosections collected at 7 days after injection. BRN3A‐positive cells and β‐III‐TUBULIN fluorescence intensity were quantified using ImageJ software (scale bar = 50 μm; *n* = 6; *p < 0.05 compared with the saline group). (c) Representative images showing the levels of BRN3A and β‐III‐TUBULIN in retinal whole mounts collected at 7 days after injection (scale bar = 50 μm; *n* = 6)

To compare the therapeutic effects between ROS‐responsive and nonresponsive Lips and between targeted and nontargeted nanoparticles, an in vivo RIR injury model was established as reported above. At 7 days after intravitreal injection, the number of RGCs in the RIR/saline group (BRN3A positive) was reduced to ~17% of the sham group. Treatment with the non‐ROS‐responsive and non‐TPP‐targeting Lips (Que@Lips), preserved the cell number to ~42% (Figures 5a and S4A) of the sham group. In stark contrast, treatment with Que@TPP‐ROS‐Lips preserved the cell number to ~66%. To confirm the protective effect of Que@TPP‐ROS‐Lips, the level of β‐III‐TUBULIN, a marker of RGCs, was assessed. As shown in Figure 5b, among the treatments, Que@TPP‐ROS‐Lips most strongly attenuated the RIR injury‐induced decrease in β‐III‐TUBULIN level.

In parallel, we also used immunofluorescence staining of retinal whole mounts to evaluate the levels of BRN3A and β‐III‐TUBULIN (Figure 5c). The trends in BRN3A and β‐III‐TUBULIN levels among the groups were similar to those obtained using the retinal vertical sections. Taken together, the above results indicate that Que@TPP‐Lips, an intraocular Que delivery nanoplatform, outperformed the non‐TPP‐conjugated and non‐ROS‐responsive platforms in protecting RGCs from RIR‐induced RGC loss.

### 3.6 Que@TPP‐ROS‐Lips alleviate retinal inflammation caused by RIR injury

**FIGURE 6:**
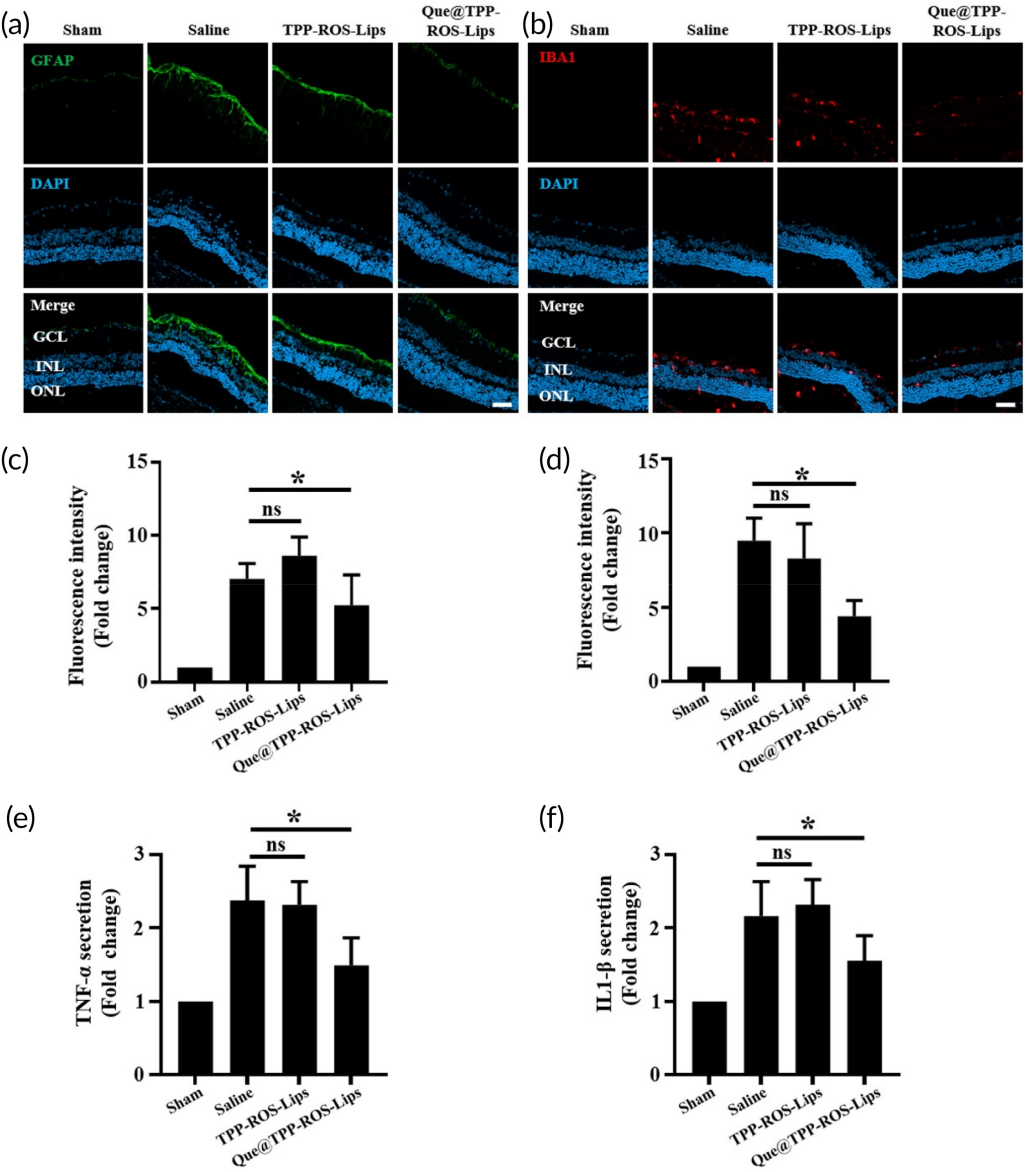
Inhibition of the RIR‐induced activation of retinal gliocytes and inflammatory cytokine secretion by Que@TPP‐ROS‐Lips following intravitreal injection. Representative CLSM images showing the levels of GFAP (a) and IBA1 (b) in retinal cryosections collected at 7 days after injection. Fluorescence intensities of GFAP (c) and IBA1 (d) quantified by ImageJ software (scale bar = 50 μm; *n* = 6; *p < 0.05 compared with the saline group). Levels of TNF‐α (e) and IL‐1β (f) determined by the ELISA. The results represent mean ± SD of fold change (scale bar = 50 μm; *n* = 6; *p < 0.05 compared with the saline group).

Inflammation is crucial in the pathological progress of RIR injury. Once stimulated by RIR, inflammatory cells such retinal microglia and macroglia (including Müller cells and astrocytes) are activated; these cells are important contributors to RGC loss.^59^, ^60^ We evaluated the activation of microglia and macroglia by measuring the levels of their markers (IBA1 and GFAP, respectively). As we hypothesized, RIR injury increased the levels of IBA1 and GFAP. Notably, Que@TPP‐ROS‐Lips effectively prevented the up‐regulation of these two proteins (Figure 6a–d). In parallel, this upregulation was confirmed by CLSM analysis of retinal whole mounts (Figure S5).

RIR injury increases the secretion of inflammatory factors including TNF‐α and IL‐1β.^5^ Thus, we assessed the expressions of these two cytokines in retinal tissues after intravitreal injection of Que@TPP‐ROS‐Lips using ELISA. We found that RIR injury increased the levels of TNF‐α and IL‐1β by approximately 2.37‐ and 2.16‐fold, respectively, compared with the sham group (Figure 6e,f). In the Que@TPP‐ROS‐Lips group, the levels of TNF‐α and IL‐1β were only 1.49 and 1.56 times those of the sham group, respectively (Figure 6e,f). Overall, these results demonstrate that the intravitreal injection of Que@TPP‐ROS‐Lips was effective at reducing inflammation associated with RIR injury.

### 3.7 Safety and distribution of Que@TPP‐ROS‐Lips in vivo

**FIGURE 7:**
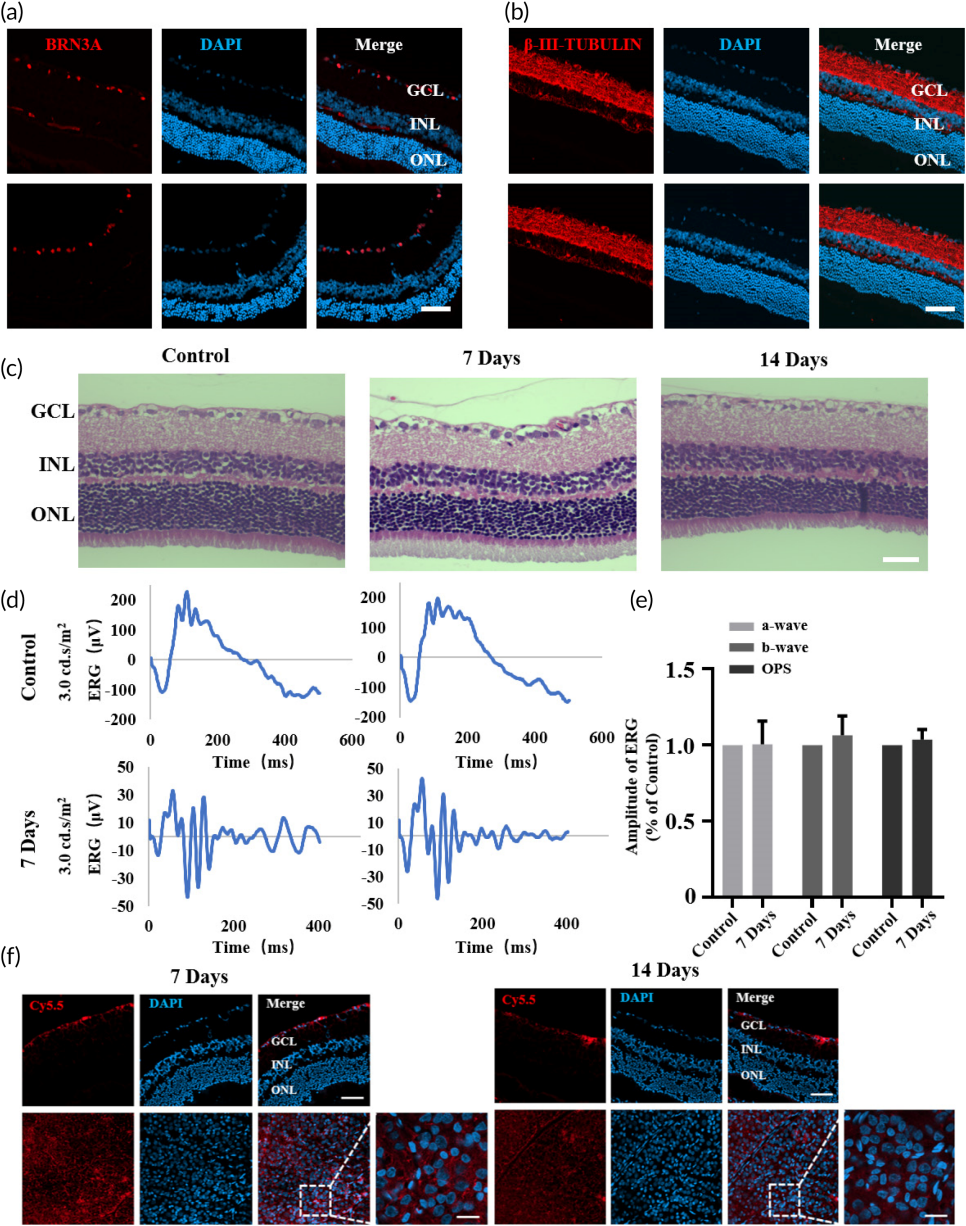
Safety evaluation and biodistribution study of Que@TPP‐ROS‐Lips *in normal rats*. After intravitreal injection of Que@TPP‐ROS‐Lips in normal rats on Days 7 and 14, retinal cryosections were subjected to BRN3A (a) and β‐III‐TUBULIN (b) immunofluorescence staining to assess retinal toxicity. Scale bar = 50 μm; n = 6. (c) H&E‐stained retina tissues collected at Days 7 and 14 after intravitreal injection of Que@TPP‐ROS‐Lips. Scale bar = 50 μm; n = 6. (d) Representative ERG traces at 3.00 cd∙s∙m^−2^ from normal rats treated with Que@TPP‐ROS‐Lips. (e) Amplitudes of the scotopic ERG a‐ and b‐wave and sums of the OPs amplitudes at the light intensity of 3.00 cd∙s∙m^−2^ (*n* = 6). (f) Localization of Que@Cy5.5‐TPP‐ROS‐Lips (red) in retinal cryosections and retinal whole mounts observed by CLSM after intravitreal injection for 7 and 14 days. Scale bar = 50 μm (upper); 20 μm (lower)

Evaluation of the safety of the nanosystem is crucial for potential clinical applications.^61^ As shown above, Que@TPP‐ROS‐Lips had no significant toxic effects in R28 cells based on analyses of cellular ATP content and LDH release (Figure 3c,d). To further demonstrate the safety of the liposomes in vivo, normal rats were intravitreally injected with therapeutic doses of the Que@TPP‐ROS‐Lips and evaluated after 7 and 14 days. The immunofluorescence results showed no significant changes in retinal RGCs until 14 days after administration (Figure 7a,b). In addition, the H&E staining images did not indicate any significant differences in retinal thickness or the number of cells in the ganglion cell layer, IPL, inner nuclear layer, and outer nuclear layer (Figure 7c). To further examine the effect of Que@TPP‐ROS‐Lips on retinal function, we performed ERG detection after intravitreal injection of Que@TPP‐ROS‐Lips in normal rats. Results showed that Que@TPP‐ROS‐Lips did not adversely affect retinal function in normal rats (Figure 7d,e). In addition to physiological conditions, we also investigated the toxicity of Que@TPP‐ROS‐Lips under pathological conditions (with RIR injury). As shown in Figure S6A,B, the contents of glutamic aspartate transaminase and alanine aminotransferase in plasma were within the normal ranges, without intergroup differences. Moreover, the H&E staining images of the main visceral organs (heart, liver, spleen, lung, and kidney) showed that treatment with Que@TPP‐ROS‐Lips did not cause noticeable histological changes (Figure S6C). Taken together, these results indicate that Que@TPP‐ROS‐Lips has no obvious toxic effects in vivo.

After observing the functional neuroprotection provided by Que@TPP‐ROS‐Lips in the ischemic retina without apparent toxic effects, we assessed the distribution of Que@TPP‐ROS‐Lips in the retina. As shown in Figure 7f, Que@Cy5.5‐TPP‐ROS‐Lips were primarily localized in the ganglion cell layer and persisted in the retina for at least 2 weeks after intravitreal injection. This is congruent with previous findings of liposomes^62^, ^63^ and suggests that Que@TPP‐ROS‐Lips can transport, in a sustained‐release manner, the hydrophobic natural product Que to the retina to exert its effect.

#### 3.7.1 Que@TPP‐ROS‐Lips up‐regulate the SIRT1/FOXO3A pathway and inhibits p38 activation in OGD in vitro and RIR injury in vivo

**FIGURE 8:**
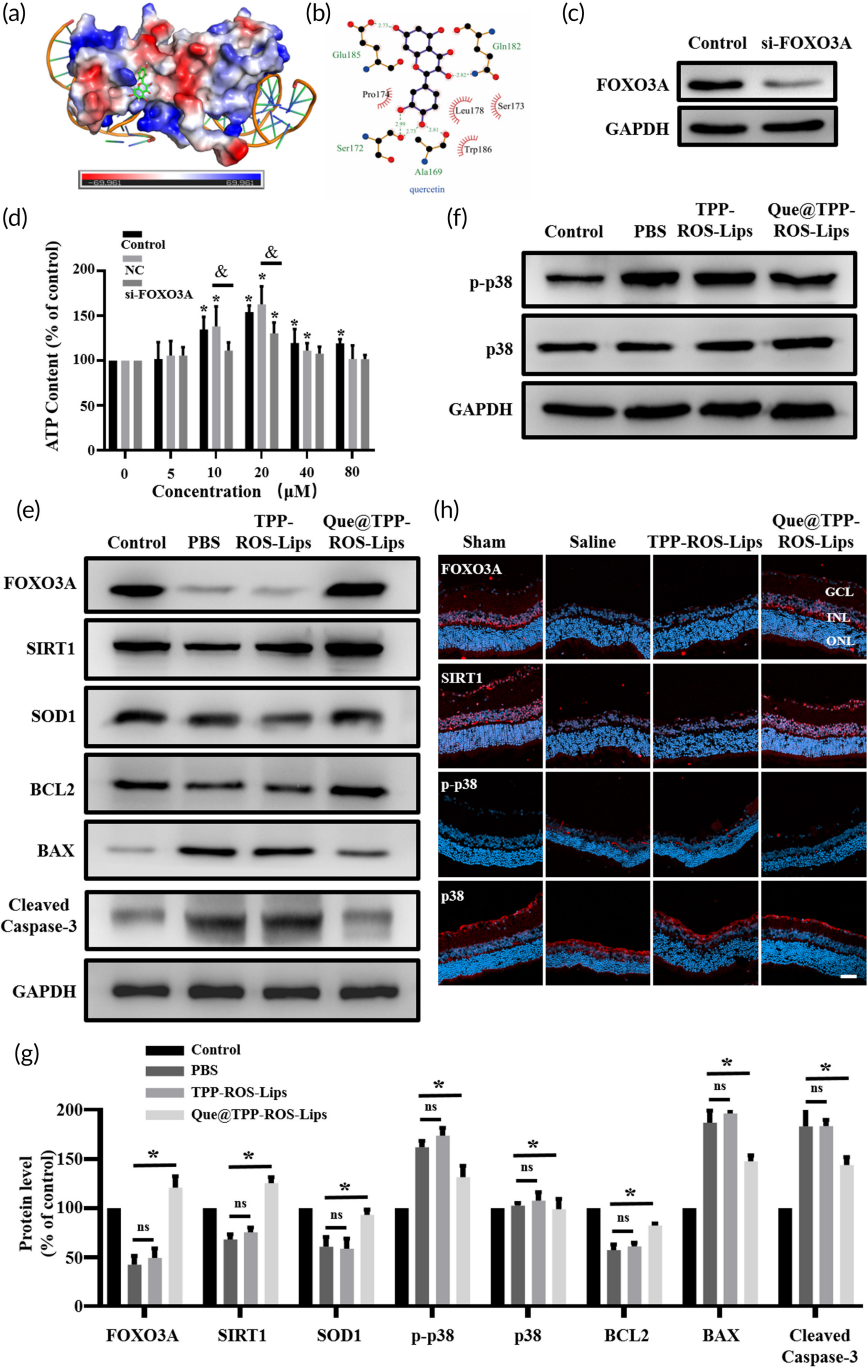
Quercetin exerts anti‐oxidative and anti‐inflammatory activity by regulating the SIRT1/FOXO3A and p38 signaling pathways. (a) Molecular docking results showing Que in the FOXO3A binding site. Que is shown as a green stick; the protein surface is colored by its electrostatic potential from red (−69.961) to blue (69.961). (b) Hydrogen bonds (green dashed lines) and hydrophobic interactions (red “eyelashes”) shown using LIGPLOT v2.2.4. (c) The knockdown effect of FOXO3A in R28 cells. (d) Cell viability (evaluated by ATP content) with or without the knockdown of FOXO3A. Data are presented as mean ± SD (*n* = 3); *p < 0.05 compared with the control group. (e, f) Representative western blots for FOXO3A, SIRT1, SOD1, BCL2, BAX, cleaved Caspase 3, p‐p38 and p38 in R28 cells with or without Que@TPP‐ROS‐Lips after OGD. GAPDH was used as the loading control. (*n* = 3). (g) The bar graphs represent the of Western blots illustrating the significant Que@TPP‐ROS‐Lips mediated amelioration of OGD‐induced changes in FOXO3A/SIRT1 pathway proteins (FOXO3A, SIRT1, and SOD1), p38 pathway proteins (p‐p38 and p38) and apoptosis‐associated proteins (BCL2, BAX, and Cleaved Caspase 3) in R28 cells treated with or without Que@TPP‐ROS‐Lips. (h) Representative immunofluorescence images of FOXO3A, SIRT1, p‐p38, and p38 in retinal cryosections indicating that Que@TPP‐ROS‐Lips‐mediated enforcement in levels of FOXO3A and Sirt1 and reduction in the level of p‐p38 in ischemic retina compared to saline group. (scale bar = 50 μm; *n* = 6). *p < 0.05 compared with the control group.

Identifying therapeutic targets is essential for the design and development of new drugs. The target and mechanism of action of Que in RIR injury have not been elucidated, hindering the application of Que in the treatment of retinal diseases. To explore the underlying mechanism of Que@TPP‐ROS‐Lips in the treatment of RIR injury, molecular docking was performed to screen therapeutic targets of Que. Que was docked into the binding site of FOXO3A (Figure 8a,b). FOXO3A, a transcription factor, has been reported to reduce oxidative stress in ischemia–reperfusion models by enhancing the activity of superoxide dismutase.^25^ The phenyl group of Que was located at the hydrophobic pocket formed by the Ala169, Pro174, Leu178, and Trp186 residues and formed multiple hydrophobic interactions (Figure 8b). Que was also firmly held in the binding site of the target by a network of hydrogen bonds with key residues including Ala169 (bond length: 2.81 Å), Ser172 (bond lengths: 2.73 Å and 2.99 Å), Glu185 (bond length: 2.73 Å), and Gln182 (bond length: 2.82 Å). The docking results were validated by silence of FOXO3A at the cellular level. FOXO3A siRNA was employed to knock down FOXO3A in R28 cells to verify the molecular docking results and assess the interactions of Que. The decrease in cell viability in the OGD model was ameliorated by Que@TPP‐ROS‐Lips. However, after the knockdown of FOXO3A, the addition of Que@TPP‐ROS‐Lips did not improve the viability of R28 cells in the OGD model, indicating a direct interaction between Que and FOXO3A (Figure 8c,d). Notably, Que@TPP‐ROS‐Lips reversed the OGD‐induced decrease in FOXO3A expression along with the decreases in SIRT1 and SOD1 expression (Figure 8e). This finding is consistent with the previously reported regulation of SIRT1/FOXO3A, a pathway that down‐regulates oxidative stress, by Rg3 in cardiac ischemia.^64^ This finding was confirmed by our results in the in vivo RIR injury model (Figure 8e). Since the results of TUNEL staining showed that Que@TPP‐ROS‐Lips can attenuate the apoptosis of retinal cells caused by RIR injury, we also detected apoptosis‐related proteins in the OGD model. We found that the cleaved form of cleaved Caspase‐3 was markedly decreased at 20 μm Que@TPP‐ROS‐Lips in treated R28 cells and Que@TPP‐ROS‐Lips increase in the expression of the anti‐apoptotic proteins (BCL2) as well as decrease the level of the pro‐apoptotic protein BAX (Figure 8e,g), indicating that OGD‐induced apoptosis is partially rescued by Que@TPP‐ROS‐Lips. Taken together, Que@TPP‐ROS‐Lips activated the FOXO3A antioxidant stress‐signaling pathway in vitro in an OGD model and in vivo in an RIR injury model. This is the first report of the antioxidant mechanism of Que in retinal ischemia.

RIR injury activates the p38 signaling pathway, and inhibition of the p38 signaling pathway can attenuate ischemic injury^65^, ^66^, ^67^ Thus, we investigated whether Que@TPP‐ROS‐Lips regulates p38 activation in RIR injury. In an OGD model, R28 cells were treated with Que@TPP‐ROS‐Lips, and the activation of p38 was monitored. Elevated p38 activation was observed in the OGD model, and this elevation was attenuated by the addition of Que@TPP‐ROS‐Lips (Figure 8f,g). As expected, the in vivo data were consistent with the in vitro data, as evidenced by immunofluorescence staining to detect p38 phosphorylation (Figure 8h). Taken together, these data suggest that Que released from Que@TPP‐ROS‐Lips targets FOXO3A to suppress oxidative stress caused by RIR injury. In addition, the Que‐mediated inhibition of p38 activation may also have contributed to this effect. We therefore infer that our targeted liposomes for efficient delivery of Que at least partially inhibited RIR injury‐induced p38 activation, which in turn directly or indirectly prevented oxidative stress and inflammation. In future studies, we will continue to explore the relationships between p38 activation, the SIRT1/FOXO3A signaling pathway, and ROS in RIR injury.

## 4 CONCLUSION

In this study, we (1) engineered the first ROS‐responsive, TPP‐targeting intraocular delivery system (Que@TPP‐ROS‐Lips) that accumulates in the retina and is safely and easily taken up by retinal cells; (2) applied this nanoplatform for Que delivery in an in vitro model of OGD and an in vivo model of RIR injury; and (3) demonstrated that the anti‐oxidant and anti‐inflammatory mechanisms of Que@TPP‐ROS‐Lips are associated with the up‐regulation of the SIRT1/FOXO3A pathway and the down‐regulation of the p38 pathway. To fully realize the potential of this drug delivery system, it is necessary to test it in future studies in chronic models of RIR injury related to diseases such as glaucoma and DR. The further development and optimization of this unique drug delivery nanoplatform may lead to a viable natural product‐based treatment for RIR injury‐associated ocular diseases.

## AUTHOR CONTRIBUTIONS

**Laien Zhao:** Investigation (lead); methodology (lead); writing – original draft (lead). **Longbing Ling:** Investigation (lead); methodology (lead). **Jing Lu:** Methodology (lead); software (lead). **Feng Jiang:** Methodology (lead); software (lead). **Jianchao Sun:** Methodology (equal); visualization (equal). **Zhen Zhang:** Methodology (equal); visualization (equal). **Yanmei Huang:** Methodology (equal); validation (equal). **Xiaoqian Liu:** Methodology (supporting); software (supporting). **Yanjuan Zhu:** Software (supporting); validation (supporting). **Xiaoxuan Fu:** Data curation (equal); visualization (supporting). **Shengjun Peng:** Validation (supporting). **Wenze Yuan:** Methodology (supporting); validation (supporting). **Ruikang Zhao:** Data curation (supporting); validation (supporting). **Zhuhong Zhang:** Funding acquisition (lead); supervision (lead); writing – review and editing (lead).

## CONFLICT OF INTEREST

The authors declare that they have no conflict of interests.

## Supporting information


**Appendix S1:** Supporting Information

## Data Availability

All data to support the findings from this study will be made available to interested investigators. The datasets used and/or analyzed during the current study are available from the corresponding author upon reasonable request.
