# Supplementary material for: Reactive oxygen species‐responsive mitochondria‐targeted liposomal quercetin attenuates retinal ischemia–reperfusion injury via regulating SIRT1/FOXO3A and p38 MAPK signaling pathways
Source: Bioeng Transl Med. 2022 Dec 1;8(3):e10460. doi: 10.1002/btm2.10460 (PMC10189480; doi:10.1002/btm2.10460)
Supplement: Supplementary file 1 — Appendix S1: Supporting Information [file BTM2-8-e10460-s001.docx]

Supplementary information


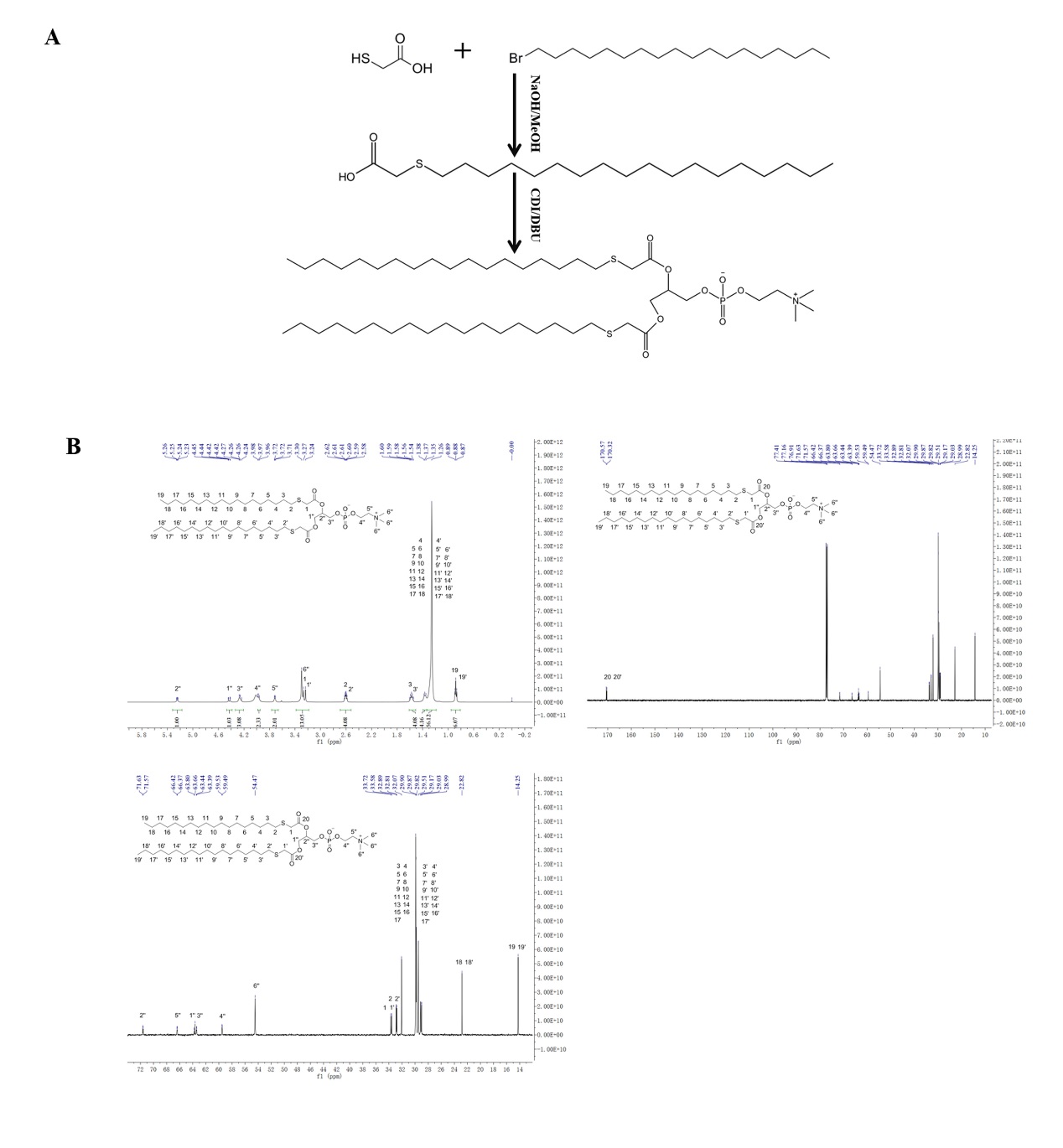


**Figure S1. Synthesis and characterization of Di-S-PC.** (A) Synthetic route of Di-S-PC lipids. (B) ^1^H NMR and ^13^C NMR (500 MHz, CDCl3) spectrum of ROS-responsive Di-S-PC lipids.


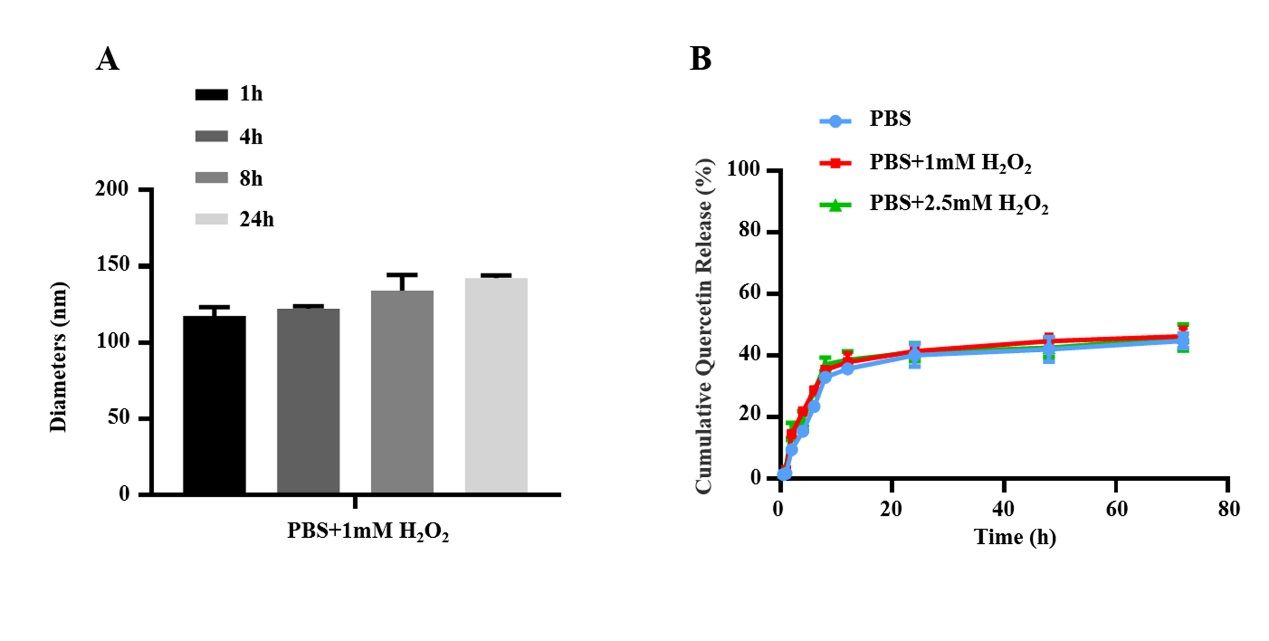


**Figure S2. Size change and drug release of Que@TPP-Lips.** (A) Size change in PBS containing 1mM H_2_O_2_. (B) Release profiles of Que@TPP-Lips in the presence of 0, 1, and 2.5 mM H_2_O_2_. Data are presented as mean ± SD (*n* = 3).


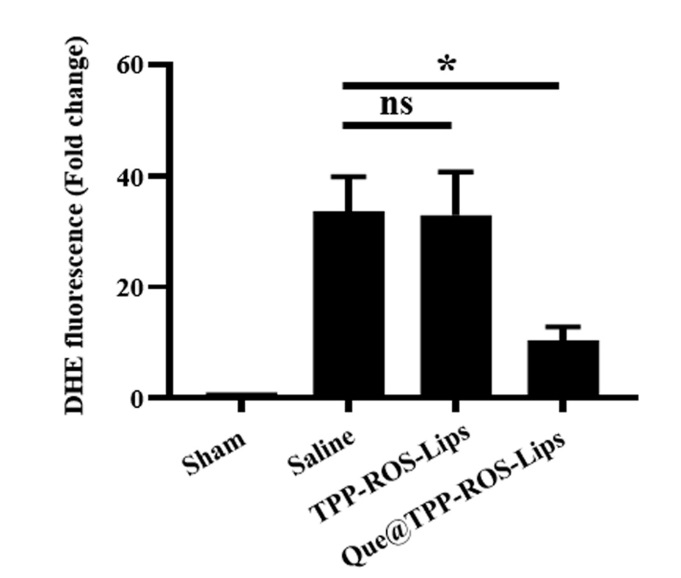
**Figure S3. Quantification of DHE fluorescence.** Data are presented as mean ± SD (n = 6); *p < 0.05 compared with the saline group.


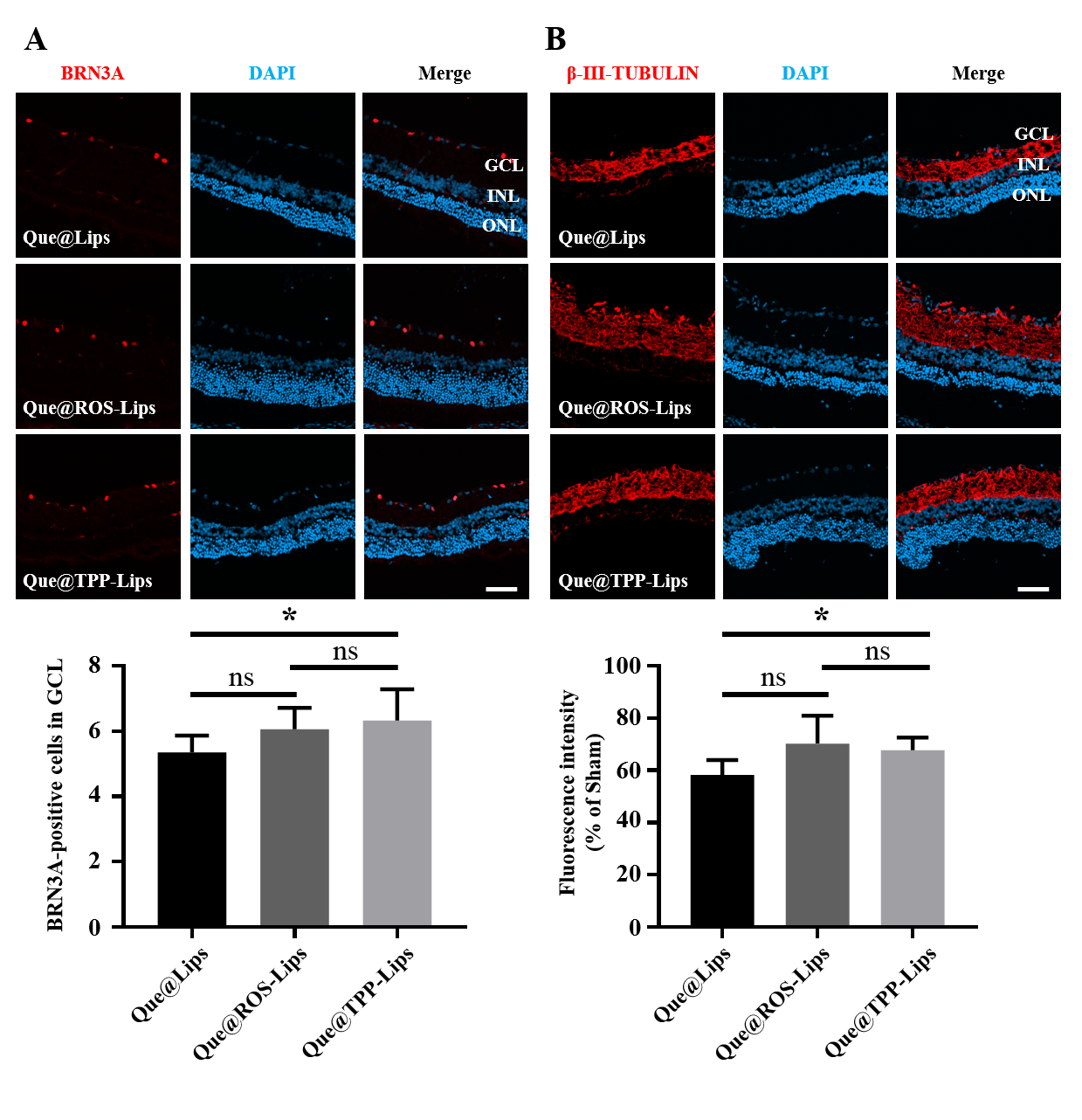


**Figure S4. Effects of different Que liposomes on the injury of RGCs induced by RIR injury.** Representative CLSM images and statistical results of BRN3A (A) and β-III-tubulin (B) fluorescence.


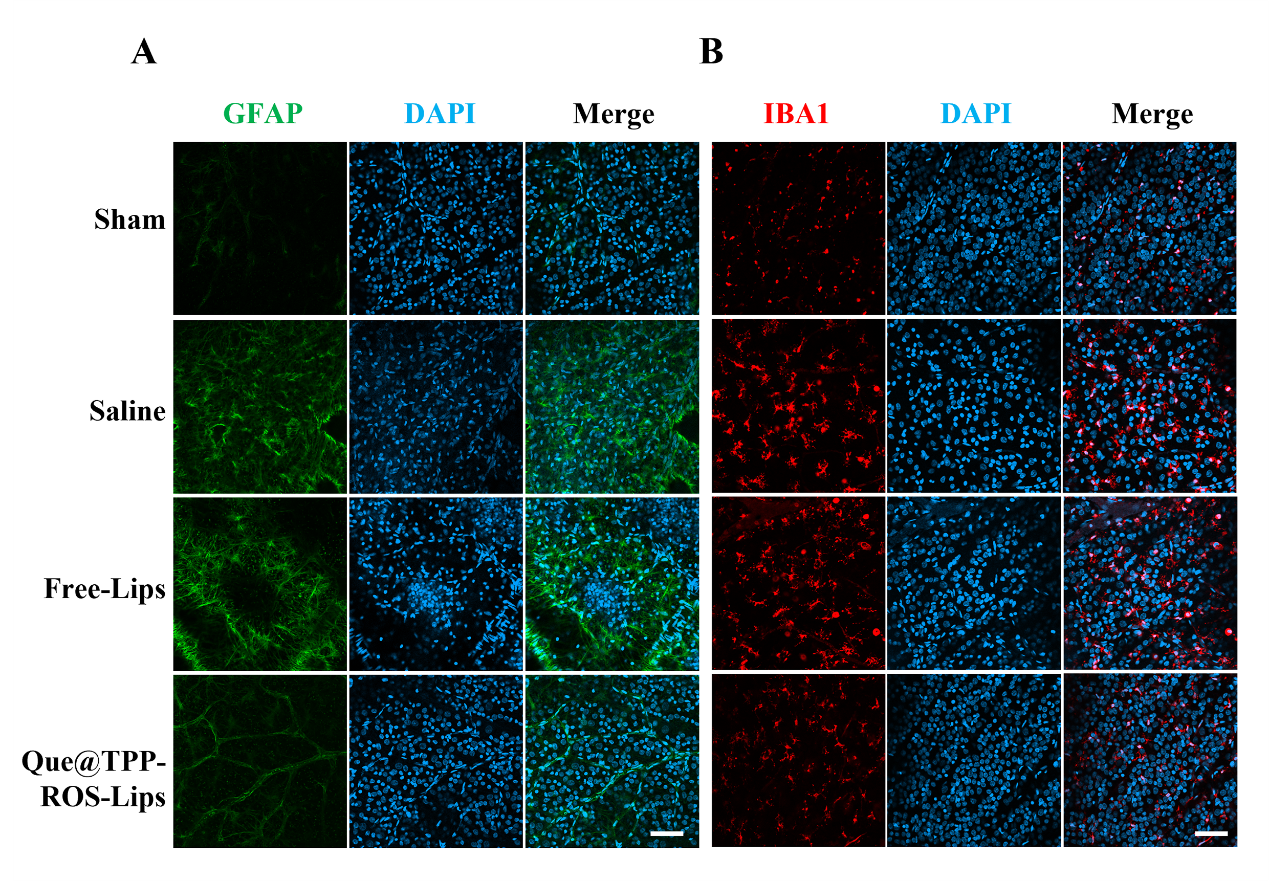


**Figure S5. Que@TPP-ROS-Lips inhibit activation of retinal glial cells.** Representative images showing IBA1 (A) and GFAP (B) proteins of retinal whole mounts collected at 7 days after injection of Que@TPP-ROS-Lips groups and other groups. Scale bar = 50 μm; n =6.


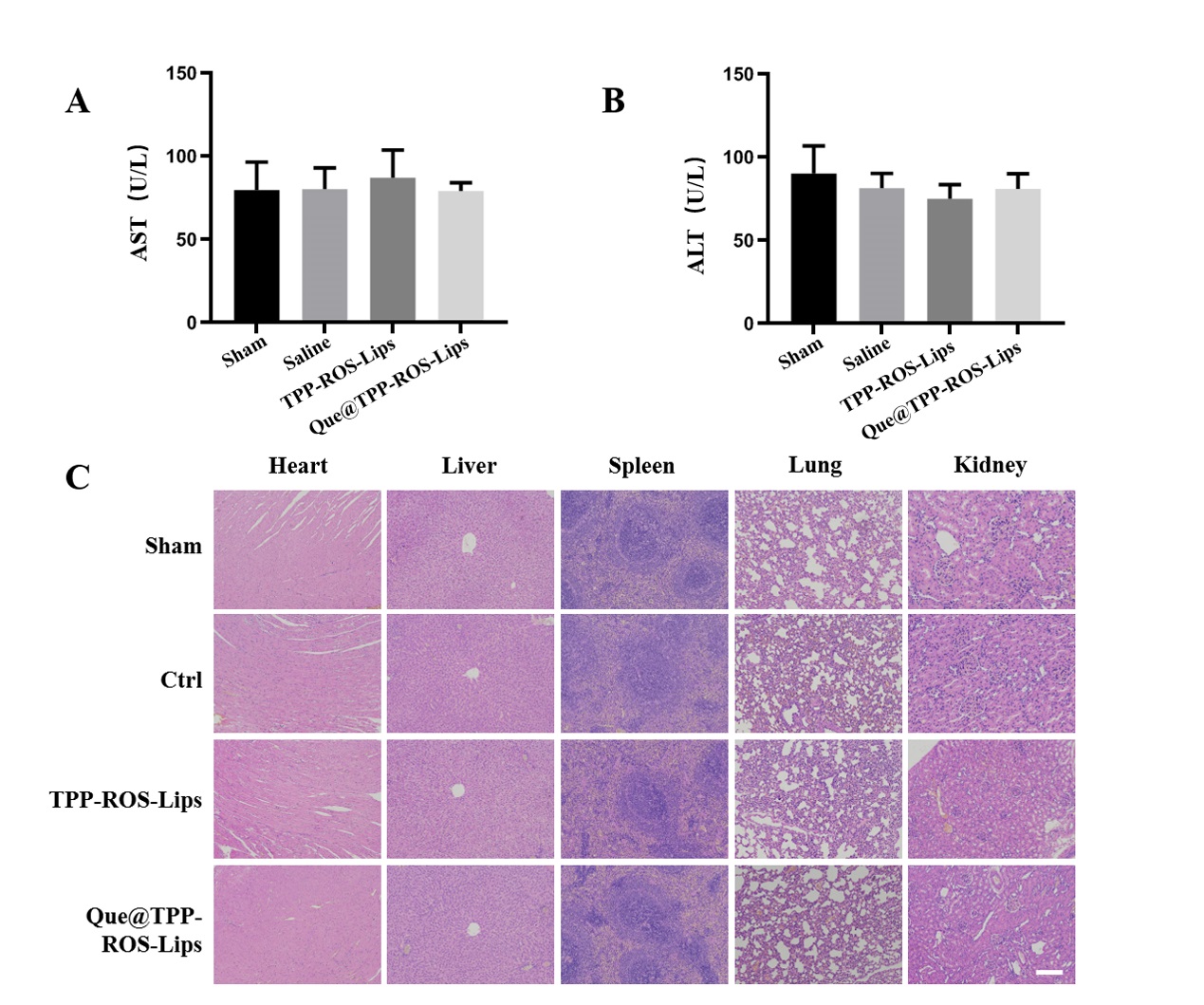


**Figure S6. Assessment of organ toxicity in** **Que@TPP-ROS-Lip-treated rats.** Measurement of serum aspartate aminotransferase (AST)（A）and alanine aminotransferase (ALT) (B) activity in rats, including groups of Sham, Sham/Saline (ctrl), TPP-ROS-Lips, and Que@TPP-ROS-Lips. (C) H&E staining of main organs; scale bars; 50 μm; n = 6.
